# A post-quantum verified self-healing framework for healthcare IoT systems: deep anomaly detection, severity estimation and risk-aware rollback to trusted checkpoints

**DOI:** 10.3389/frai.2026.1913803

**Published:** 2026-08-26

**Authors:** Arul Treesa Mathew, Prasanna Mani

**Affiliations:** School of Computer Science Engineering and Information Systems, Vellore Institute of Technology, Vellore, Tamil Nadu, India

**Keywords:** anomaly detection, checkpoints, deep learning, healthcare IoT systems, post-quantum verification, rollback

## Abstract

Stronger defense mechanisms are needed for the safety of healthcare IoT systems, for faster detection and smooth recovery from cyber-attacks, without disrupting services. This study addresses the need to safeguard healthcare IoT systems through efficient detection of potential anomalies leveraging the capacities of deep learning, and also rolling back the system to the safest checkpoint in the recent past, verified using post-quantum verification using ML-DSA/CRYSTALS-Dilithium. A two-tier architecture is proposed in this study with deep anomaly detection and attack severity analysis in the first tier, and post-quantum verification for selecting a policy-driven safe rollback point based on multi-factor assessment, with due consideration of risks. The reinforcement learning-based framework is used in the checkpoint selection phase that selects the checkpoint with the best Q-Score. The model is implemented over two prominent HIoT network datasets–WUSTL EHMS 2020 and CIC IoMT 2024. The model is evaluated using anomaly detection metrics (attack probability, confidence score, severity analysis, and recovery uptime), trust score, verification latency, recovery time, rollback quality, and performance metrics (F1-score, false-positive rate, precision, and recall). The proposed model achieved strong overall performance across both healthcare IoT datasets, with average values of 59.23% attack probability, 94.00% confidence, 5.61% anomaly score, 69.50% severity, 26.61 ns recovery time, 75.86% trust score, 98.37% uptime, 72.93% rollback quality, 11.28 ns verification latency, 98.37% F1-score, 98.28% recall, 98.4% precision, and 0.045% false positive rate. The proposed model effectively and accurately detects an attack and proposes the suitable recovery method–micro rollback, medium rollback, or complete rollback- based on the attack severity analysis. Better system uptime is ensured by selecting recent and trustworthy checkpoints to roll back based on post-quantum verification and combined trust score calculation.

## 1 Introduction

The quality of service offered by the healthcare domain has improved exponentially following the introduction of Healthcare Internet of Things (HIoT), which incorporates smart devices that help in efficient monitoring, analysis, and data-driven, accurate clinical decisions. Wearable monitors, bedside assistant devices, implants, and device-to-cloud analytical platforms make decision-making accurate and efficient. Despite these operational efficiencies, the popularity and extensive use of these smart systems have also led to increased attacker interest, owing to the sensitivity and personalized behavior of the contained data. Attacks mostly target disrupting the integrity and confidentiality of the contained data, as well as the continuity of service offered by the system. Attacks aimed against HIoT systems increase annually at a rate of 18% (Khalil, 2025) according to recent studies. This alarms the need to safeguard the HIoT systems, making them both resilient and recoverable upon the occurrence of an attack.

Attacks against healthcare IoT systems (Luo et al., 2026) are mostly initiated from a set of compromised devices in the target network. These devices are used to launch attacks against victims, leading to either a data breach or denial of service. The fundamental security considerations of confidentiality, integrity, and availability are violated upon the occurrence of a successful attack. Since healthcare IoT systems are deployed to ensure continuous service for stakeholders, disruption cannot be tolerated beyond a specific level. Downtime must be minimized even in the event of an adversarial action. Recovering sensitive systems (Bisola et al., 2025) like healthcare IoT requires rollback checkpoints that can mitigate risks encountered during the process.

Even though strong security protocols (Naghib et al., 2025), continuous security monitoring, regular updates, and a constant response plan are required to mitigate attacks on HIoT systems, conventional systems often fail to provide an efficient method to address this. Enhancements using artificial intelligence (Baba et al., 2025) and deep learning (Wankhade and Sharma, 2025) have improved detection speed, but have not yet addressed recovery after a successful intrusion. Intrusion detection systems operate at three levels of the network: the device level, fog level, and cloud level. When designing an efficient intrusion detection system (Siqueira et al., 2025) for HIoT, proper consideration should be given to data-related, deployment-related, model-related, and operational challenges.

Conventional techniques for intrusion detection and recovery in HIoT systems operate in a specific mode, either focusing on intrusion detection or on recovery. This results in higher false alarms and higher downtime for service continuity, owing to slower rollback decisions. Common encryption techniques are based on RSA or elliptic-curve cryptography, which fail against quantum-capable adversaries. The integrity of stored checkpoints that are selected based on previous trust scores will be under threat in such circumstances. The main motivation of this study is these limitations of the conventional systems, thus leading to the design of an integrated approach that can couple faster anomaly detection with efficient rollbacks to post-quantum-verified trustworthy checkpoints.

**Figure 1:**
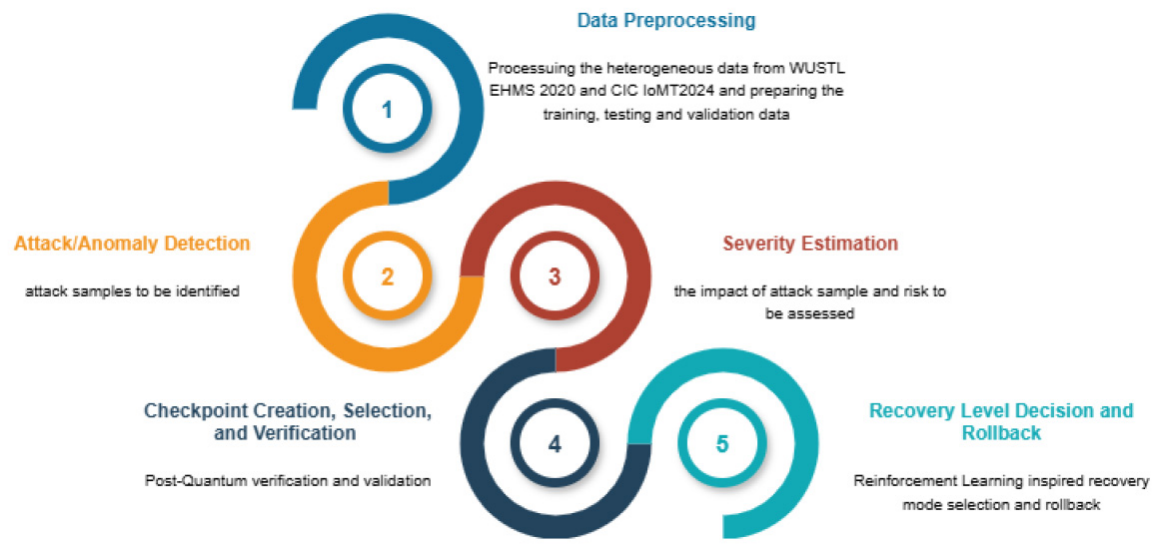
Steps in two-tier deep anomaly detection and post-quantum verified risk-aware rollback model.

The proposed model operates on a two-stage pipeline: a hybrid classifier encoder for deep anomaly detection and attack severity estimation in the first stage, followed by identification of a trustworthy checkpoint for risk-aware rollback using the Dilithium verifier, and policy learner-based decision-making for rollback based on attack intensities. The model is experimented over two common datasets for HIoT network data: WUSTL EHMS 2020 (Hady et al., 2020) and CIC IoMT 2024 (Dadkhah et al., 2024). The model's performance on the two datasets is evaluated. It demonstrates improved recovery time, trust score, uptime, and rollback quality compared to static rollback baselines, while preserving high detection F1 and a low false positive rate. Figure 1 shows the main steps involved in the proposed system model.

The main contributions of this study are as follows:

- A two-stage self-healing HIoT system with resilience to future quantum-capable adversaries
- Deep anomaly detection based on a hybrid deep learning approach, using an MLP classifier and an autoencoder
- Post-quantum authenticated checkpoint signing and verification using ML-DSA/CRYSTALS-Dilithium verifier
- Trust scoring based on multiple factors affecting detection and recovery for efficient decision making
- Reinforcement Learning-based policy learner for checkpoint selection upon the event of an attack.

The rest of the article elaborates on the related literature analysis, the proposed methodology, comparative performance evaluation, analysis, and future research directions.

## 2 Related literature

The following section describes recent and related literature on existing systems for fast and accurate attack detection, recovery and rollback, and post-quantum-based verification for healthcare IoT systems.

### 2.1 Anomaly/Attack detection

Fast and effective detection of traffic that might result in a successful attack is significantly important in sensitive systems like Healthcare IoT. Various methods have been proposed to date for effective anomaly detection and analysis. Table 1 consolidates the analysis of recent techniques in practice for efficient detection of anomalies in healthcare IoT systems.

**Table 1:** Attack/Anomaly detection in Internet of Things.

Method	References	Methodology	Findings	Analysis
Intrusion Detection Systems (IDS) for modern healthcare systems	(Siqueira et al., 2025; Baba et al., 2025)	Healthcare 5.0 approaches based on fused network and biomedical features	Intrusion Detection Systems methods, importance of fusion of features, detection of complex attacks, Significance of AI based techniques focusing on explainable, privacy-preserving, low-latency IDS dimensions	Study on existing methods for IDS and the heterogeneity of their operations
HIoT: Heterogeneous information network based compromised IoT device detection	(Luo et al., 2026)	Compromised IoT device detection using Heterogeneous information network	Label propagation algorithm for detecting compromised devices, proven over real dataset	Considers only physical layer attack levels
Generative AI for IoT Security	(Wankhade and Sharma, 2025; Shanmuga Priya and Senthil, 2025; Ullah et al., 2025)	Use GANs, VAEs, and generative-AI based techniques for detection and prevention of attacks in IoT systems	Generative AI, capacities, efficient detection and prevention mechanisms using GANs, combinational approaches using other AI approaches along with Generative AI	Study on existing state-of-the-art mechanisms for attack detection using Generative AI
Auto Encoder based Detection	(Narmadha and Balaji, 2025; Zeng et al., 2025)	use autoencoders combined with other efficient models like LSTM for anomaly detection	Stable performance levels over varying datasets	Higher rates of false negatives, high reconstruction errors
Multilayer Perceptron (MLP) based Detection	(Kadiyala et al., 2025; Kushnerov et al., 2025; Saranya and Valarmathi, 2025; Zachos et al., 2025)	MLP classifier trained on labeled normal/anomaly data	Simple, fast, efficient over moderate data streams	Mostly works over labeled data

### 2.2 Self-healing approaches

Proper recovery methods (Veeravalli, 2026; Bök and Grosse-Kampmann, 2025) have to be automatically selected, and system operations should be recovered to a recent safe state, in minimal downtime, especially for sensitive systems like healthcare IoT systems. Research articles that properly align recovery and rollback using verified checkpoints for healthcare IoT systems are limited; hence, the study has also included Internet of Things-based applications from a generic perspective. Table 2 consolidates the recent techniques in practice for recovery and rollback in the Internet of Things environment.

**Table 2:** Rollback and recovery in Internet of Things.

Method	References	Methodology	Findings	Analysis
Federated learning based self-healing approaches	(Salim et al., 2025; Kumar et al., 2025)	Federated Learning combined with advanced cryptographic techniques	Focuses on faster recovery of system	Mostly suited for blockchain based implementations
Checkpoint selection for recovery	(Nellutla et al., 2025; Zekun et al., 2025)	Identification of suitable safe states from the recent past of the system	Effective recovery of system, better continuity of service	Proper verification of selected checkpoints is not explicitly taken care of
ML-based approaches for recovery	(Ram Kumar et al., 2025; Raptis et al., 2025; Naik et al., 2025)	Machine learning approaches for effective rollback decisions	Leveraging the strengths of AI-based techniques	Verification of selected checkpoints and trustworthiness are not given proper consideration

### 2.3 Post-quantum techniques for verification

The long-lived medical deployments of healthcare IoT systems make classical cryptographic verification insufficient to protect device identities, selected and encrypted checkpoints, and preserve system integrity during recovery against future quantum attacks. Hence, implementing a post-quantum verification ensures that a checkpoint remains trustworthy irrespective of a future quantum-based attack (Barna et al., 2025). Table 3 consolidates the study on various post-quantum approaches in use for IoT-based systems.

**Table 3:** Post-quantum verification techniques.

Method	References	Methodology	Findings	Analysis
Post-quantum authentication techniques	(Mansoor et al., 2025; Hanna et al., 2025; Abdullah and Atia, 2026)	Hybrid quantum algorithms for efficient authentication in distributed systems	Quantum-based techniques combined with traditional encryption and key exchange techniques to ensure resilience against future quantum-based adversaries	The potential of quantum-based adversaries is to be defined explicitly
Post-quantum secure trust management	(Khan and Khan, 2025; Vaigandla, 2025; Hussain et al., 2026)	NIST-standard Post Quantum Cryptography primitives combined with dynamic trust scoring, zero-trust policy engines, and lightweight machine-learning classifiers	Stronger levels of security from both quantum and traditional modes of attacks with usable authorization and detection levels, maintaining better performance levels as compared to classical approaches	Lack of fully adaptive trust models for heterogeneous environments like HIoT systems
Post-quantum cryptographic verification	(Saeed and Alqahtani, 2026; Sharma and Rani, 2025; Kumar et al., 2026)	Combination of NIST-standardized algorithms with formal verification tools to ensure that the system remains secure under quantum-resistant assumptions.	Post-quantum cryptographic verification techniques offer better resilience against both classical and quantum-era adversaries	Balancing the real-world applications with post-quantum-based libraries requires explicit efforts

### 2.4 Findings from literature analysis

Healthcare Internet of Things has progressed holistically in recent years. Security measures mainly consider 3 dimensions: faster and more accurate anomaly/attack detection, safe recovery with proper system rollback in minimal downtime, and stronger verification methods to enforce better resilience. However, most approaches mainly focus on one aspect of the above. Research focusing on faster and more accurate anomaly detection leaves recovery and rollback unattended. State-of-the-art techniques for efficient system restoration assume a compromised system. Most techniques in use either cope with classical attack modes and adversaries or cater to quantum-based adversaries. Combining adaptive rollback decisions with post-quantum signature-based verification for checkpoint validation will offer unified security and real-time resilience to advanced attacks. A security system that offers faster anomaly detection, severity analysis, and proper rollback selection, restoring the system to a post-quantum-verified checkpoint that is both recent and trustworthy, will help achieve better security and resilience for sensitive systems like HIoT.

## 3 Proposed system

**Figure 2:**
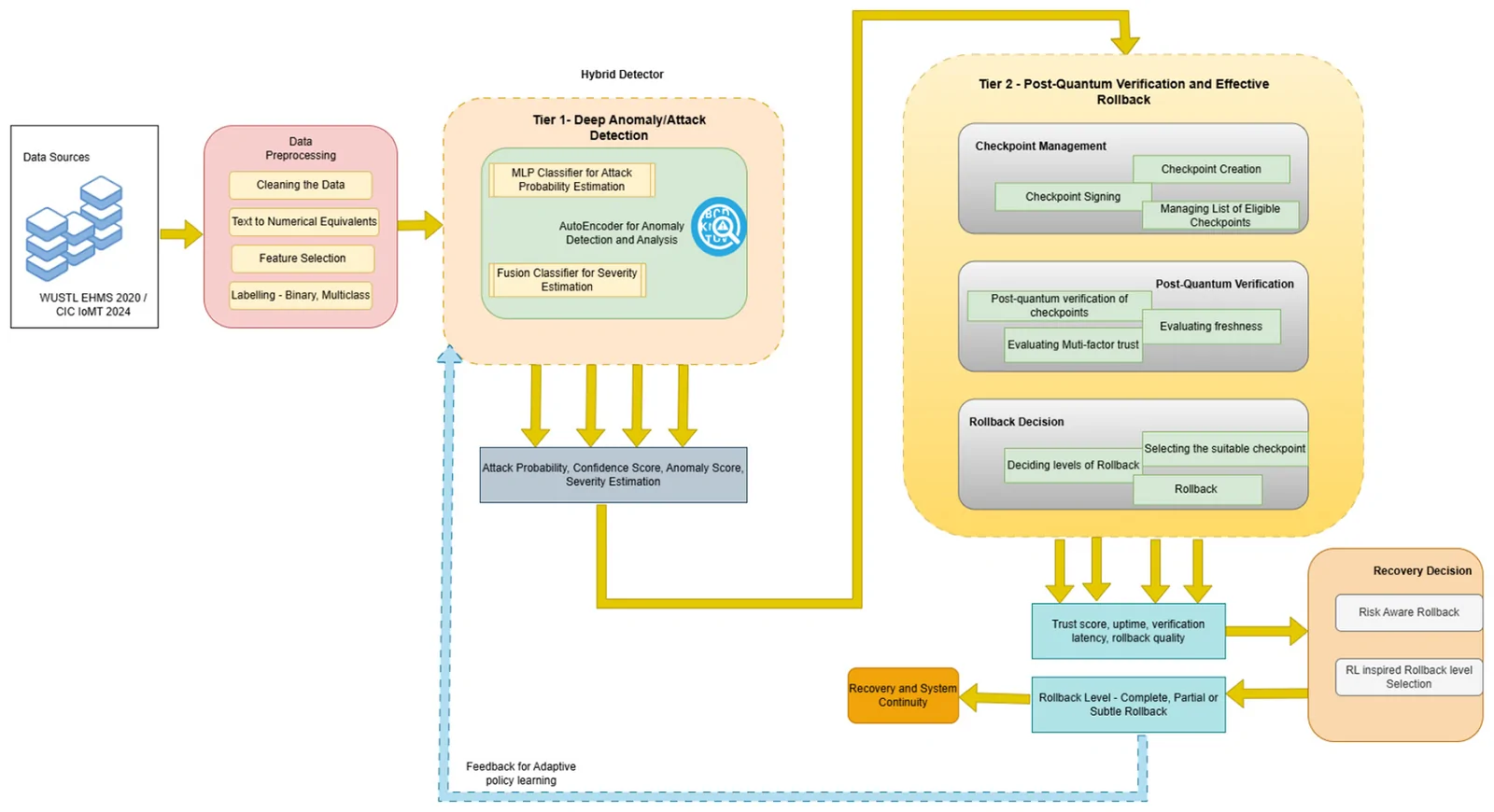
System architecture.

**Figure 3:**
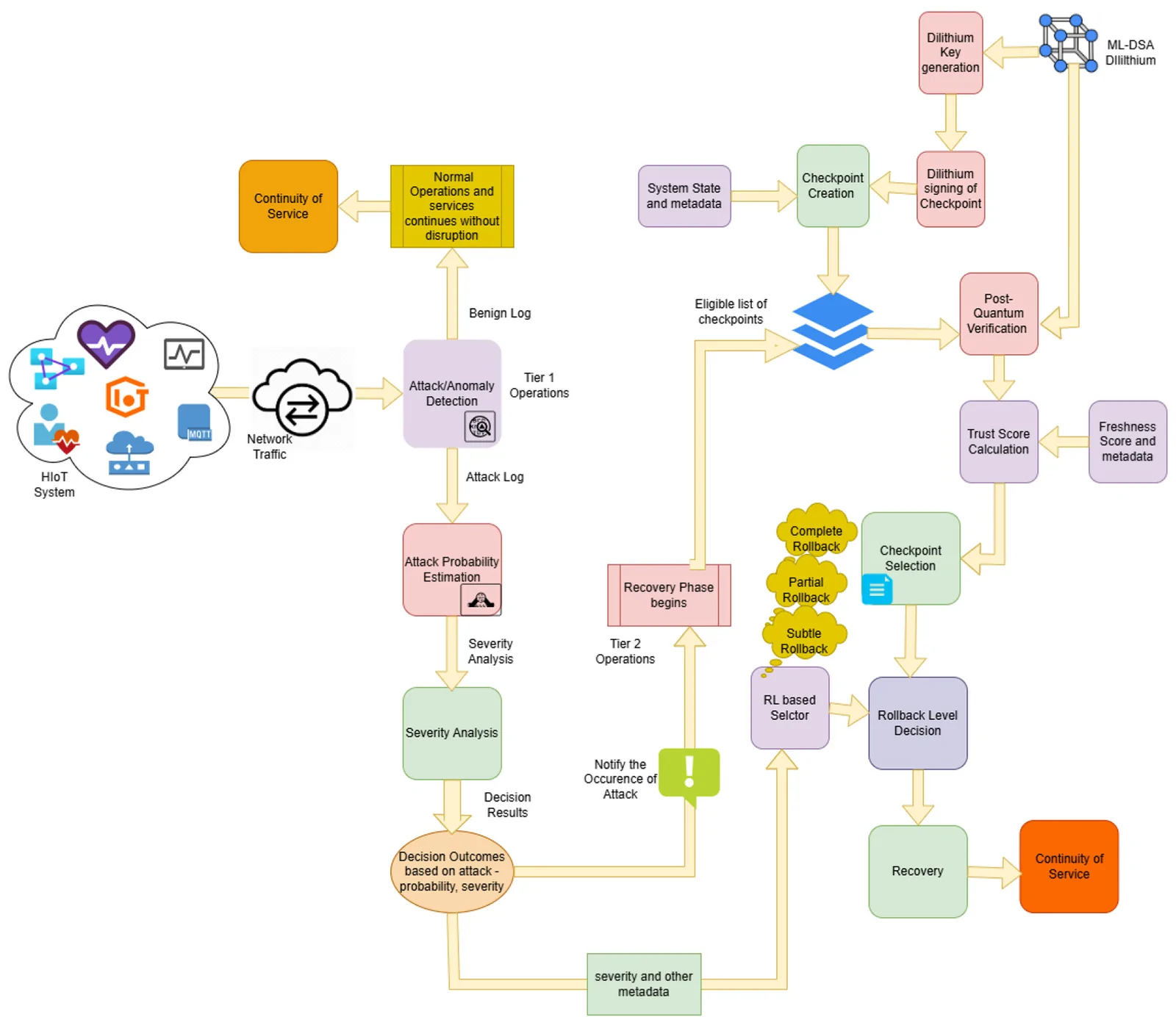
System workflow.

This section details the proposed two-stage deep anomaly detection and post-quantum risk-aware rollback system. The framework employs a post-quantum-resilient self-healing pipeline, particularly for HIoT systems. It combines deep anomaly detection, post-quantum checkpoint verification, and risk-aware rollback under an RL-inspired policy together to enforce higher levels of efficiency. Figures 2, 3 show the system architecture and detailed system workflow of the proposed model, respectively.

### 3.1 Datasets and data preprocessing

The model is implemented on two standard network datasets for HIoT systems–WUSTL EHMS 2020 and CIC IoMT 2024. WUSTL EHMS 2020 enforces a binary classification over network traffic as normal or attack traffic, whereas CIC IoMT 2024 offers multi-class classification based on the types of attacks identified.

#### 3.1.1 Dataset description

##### 3.1.1.1 WUSTL EHMS 2020

Developed by Washington University in St. Louis (WUSTL), this real-time benchmark dataset is built over medical sensors, gateways, network infrastructure, and visualization/control systems. This dataset is developed mainly to study intrusions aimed at healthcare systems. The patient-specific healthcare data collected using the testbed is transferred through the network for potential interceptions. This synthetic dataset contains over 16,000 records and is widely used for studies focused on securing healthcare IoT systems. Table 4 consolidates the features of the WUSTL-EHMS 2020 dataset.

**Table 4:** Characteristics of the WUSTL-EHMS 2020 dataset.

Aspect	Details
Data types	44 features in total 35 network flow metrics 8 patient biometric features 1 label feature (attack vs. normal)
Dataset size	~4.4 MB, stored in CSV format
Samples	Normal traffic: 14,272 (87.5%) Attack traffic: 2,046 (12.5%) Total: 16,318 samples

##### 3.1.1.2 CIC IoMT 2024

Developed by the Canadian Institute for Cybersecurity (CIC), this dataset is aimed at enabling the development and evaluation of Internet of Medical Things (IoMT) security solutions. Eighteen types of attacks were carried out on the experimental testbed, which had 25 real and 15 simulated devices. These attacks are classified into five attack groups: DDoS, DoS, Reconnaissance, MQTT, and spoofing. Table 5 consolidates the characteristics of the CIC IoMT 2024 dataset.

**Table 5:** Characteristics of the CIC IoMT 2024 dataset.

Aspect	Description
Devices	40 IoMT devices (25 real, 15 simulated)
Protocols considered	Wi-Fi, MQTT, Bluetooth
Attack types	18 attacks in five categories (DDoS, DoS, Reconnaissance, MQTT, and spoofing)
Data format	CSV (≈268 MB)
Collection method	Network tapping, malicious devices
Lifecycle profiling	Power, idle, active, interactive states
Evaluation	Machine learning models for detection/classification

#### 3.1.2 Data preprocessing

In this phase, both datasets are processed to have a common format on which the model can work. This is initiated by replacing string-based labels with numerical equivalents, without losing their class identities. The numerical feature fields are then examined to replace empty data values with zeroes. This is followed by appropriate feature selection and standardization. The StandardScaler function is used to standardize the features across both datasets, producing binary labels, multi-class labels, scaled feature matrices, feature lists, and the fitted scaler object.

Algorithm 1 explains the data pre-processing steps in detail. The raw dataset is represented as $D_{\text{raw}}={\left\{\left(v_{i},l_{i}\right)\right\}}_{i=1}^{N}$, where *v*_*i*_ is the feature vector of the *i*-th record and *l*_*i*_ is its class label. After removing non-numeric identifiers and standardizing the retained features, the final input matrix is denoted by *V* ∈ ℝ^*N*×*d*^, where *N* is the number of records and *d* is the number of retained features.

Algorithm 1 Data preprocessing for the post-quantum-resilient H-IoT self-healing framework. 
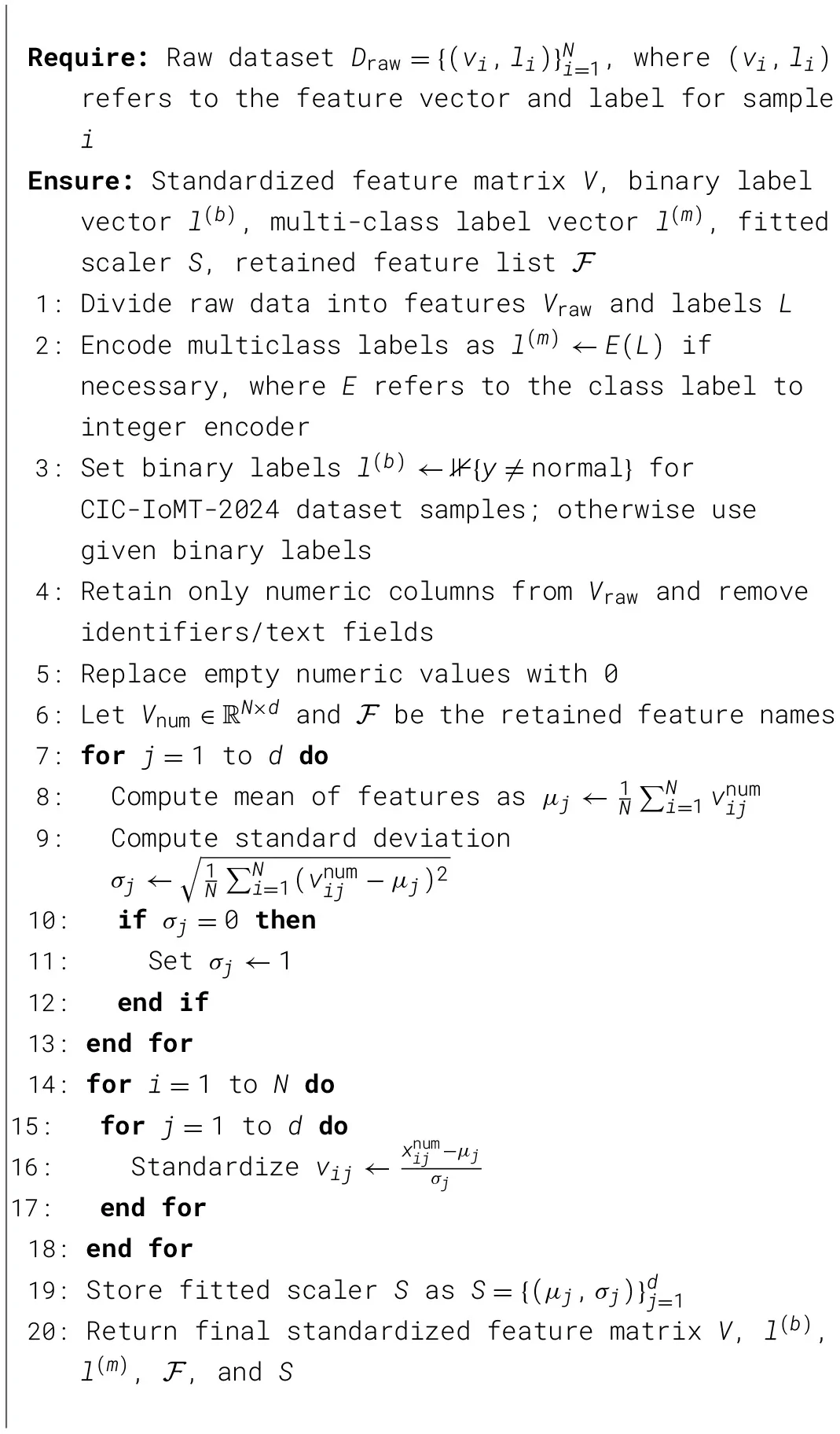

The binary and class-based labeling at this preprocessing phase helps in attack detection and its classification. Binary classification helps classify between normal and attack traffic, as mentioned in the WUSTL EHMS 2020 dataset, whereas multi-class labeling helps classify the detected attack to its type from the five different classes featured in the CIC IoMT 2024 dataset.

Standardizing selected features is important because it addresses the multi-scaled features of IoMT datasets, which cannot be optimized efficiently due to their varying magnitudes. Standardization helps with faster convergence and gradient-based learning, and it also enables efficient analysis of reconstruction error after normalizing the inputs. *Z*-score normalization is used to standardize the features.

### 3.2 Tier 1: deep anomaly detection and severity estimation

In this phase, a combined anomaly score and severity estimate are calculated based on the classifications using the MLP classifier, followed by encoding using the MLP autoencoder. The classifier is binary in nature and classifies between benign and attack traffic. Severity here refers to the intensity of deviation for the current sample from the expected normal values. The MLP autoencoder helps in modeling the anomaly based on error-based reconstruction. Algorithm 2 explains various steps involved in this tier.

Algorithm 2 Tier 1: Deep Anomaly Detection and Severity Estimation. 
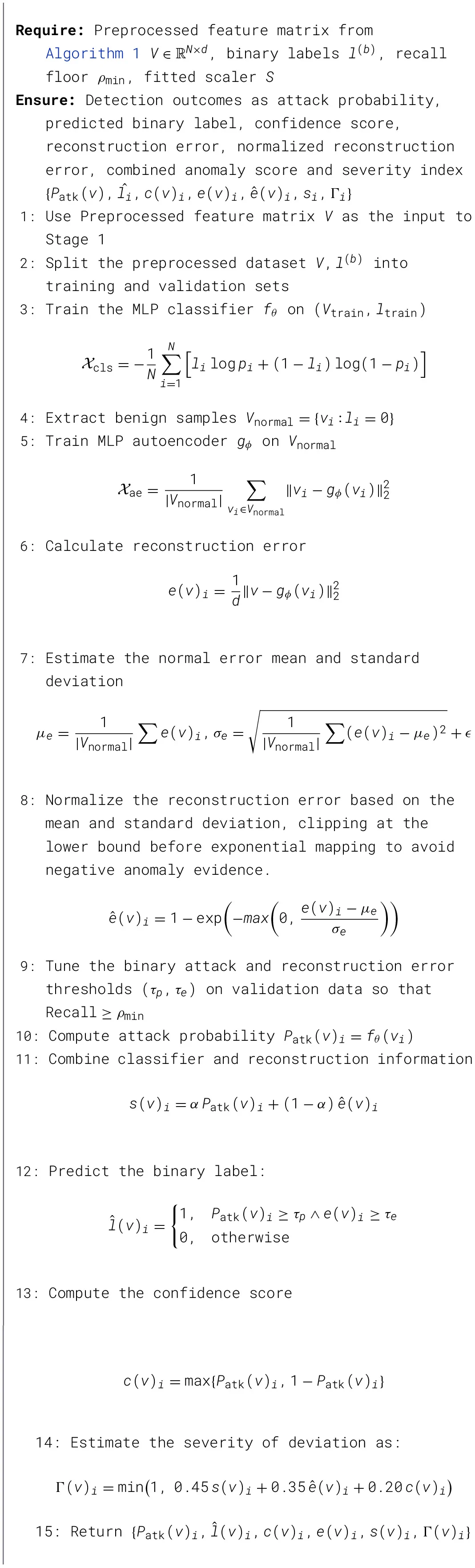

#### 3.2.1 Operational details–Tier 1

In Tier 1, we have computed various parameters that help to detect, assess, and respond to the occurrence of attacks in healthcare IoT systems.

##### 3.2.1.1 MLP classifier

The multilayer perceptron classifier used here helps to classify activity samples into attack and normal or benign classes. The classifier is trained over discriminative network-flow and biometric features, such as packet statistics, flow duration, heart rate, and oxygen saturation. Then, it detects malicious activities from the input data.

##### 3.2.1.2 MLP autoencoder

The MLP autoencoder is trained over the behavioral patterns of normal network traffic. The encoder is trained on the representations of benign traffic and physiological states, thus interpreting any deviations from normal expected behavior in the input sample upon reconstruction. Table 6 summarizes the implementation details of the MLP classifier and AutoEncoder used in the proposed model.

**Table 6:** Implementation of MLP classifier and MLP AutoEncoder.

Component	Setting
Input preprocessing	Standardization of numeric features using StandardScaler, removing null values, and converting non-numeric values to binary labels
Supervised detector	MLP classifier with hidden layers (64, 32)
Reconstruction branch	MLP autoencoder with hidden layers (32, 16, 32)
Maximum iterations	350 for both MLP classifier and autoencoder
Random seed	42
Early stopping	In use for the classifier
Data split	56% training, 14% validation, 30% test
Activation function	Scikit-learn MLP with ReLU hidden activations
Optimizer	Adam Optimizer default from scikit-learn
Threshold tuning	Validation-based sweep with a recall floor of 0.95

##### 3.2.1.3 Reconstruction error

Reconstruction error refers to the level of deviation for an H-IoT record from normal system behavior. Lower reconstruction error values indicate greater proximity to normal healthy operations, whereas higher values indicate abnormal network activity or even compromised physiological network traffic. The sensitive nature of healthcare systems, owing to the personalized version of contained data, makes it extremely important, even if the reconstruction error is minimal.

##### 3.2.1.4 Combined anomaly score calculation

In this study, the classifier probability in detecting an attack sample is combined with the reconstruction error to derive the combined anomaly score. This is important because it provides information on both the occurrence and the type of attack detected. The classifier-based attack probability is significant when attack patterns conform to known malicious patterns, whereas the autoencoder-based score identifies deviations and their intensities that might not be effectively identified at the classifier level. This combined anomaly estimation is efficient for handling the heterogeneous nature of healthcare systems, considering the occurrence of both network-based and biometric data-based anomalies.

##### 3.2.1.5 Severity estimation

Severity estimation is of prime significance in this study, as the final calculation is based on the confidence score of the classifier, attack probability, reconstruction error, and a preset combination weight. This estimation helps in assessing the impact of the attack, which will be utilized in the subsequent tier for efficient rollback decisions.

### 3.3 Tier 2: post-quantum-based checkpoint verification and risk-aware rollback

In this phase, a risk-aware self-healing healthcare system is being implemented based on the outputs from Algorithm 2. Here, Risk-aware refers to the fact that the system treats all anomalies not with a uniform preset approach, but based on their severity, classifier confidence score, checkpoint validity, and verification latency. This multi-factor-based assessment helps in efficient risk assessment of the attack situation, thereby deciding the best possible recovery rollback. This is significant in sensitive systems like healthcare, as recovery should not lead to extra overhead when the attack event is lower in severity. Self-Healing in this context refers to recovery and effective rollback to safe and verified checkpoints automatically. In this study, a post-quantum-resilient verification for a safe checkpoint is done, and the appropriate checkpoint is selected to which the system can roll back to safety.

#### 3.3.1 Post quantum digital signature algorithm ML-DSA/CRYSTALS DiLithium

CRYSTALS-Dilithium post-quantum digital signature algorithm has been standardized and renamed ML-DSA by NIST (2024) in FIPS 204. The security offered is based on module-lattice problems, like Short Integer Solution(SIS), where the goal is to find the shortest integer that can solve the lattice problem, and Learning With Errors (LWE), where the security comes from adding a small noise to the original message, which makes it difficult to decrypt without the actual secret key (Aakash et al., 2026). This approach is hence believed to be resilient against both classical and quantum adversaries. ML-DSA includes signature generation and verification functionalities that provide message integrity, authenticity, and non-repudiation (İnce, 2026). The main advantage of this signature mechanism is that the keys generated are larger than those of classic cryptographic algorithms like RSA or ECDSA, but still faster to compute. The large key size has to be addressed properly while using ML-DSA, but the enhanced security it offers, even against post-quantum adversaries, makes it acceptable. Table 7 summarizes the relevance of ML-DSA in the proposed study based on various performance metrics.

**Table 7:** Relevance of ML-DSA in the proposed checkpoint-verification workflow.

Property	ML-DSA performance	Implication
Security strength	Strong	Longer protection for verified checkpoints
Verification speed	Efficient	Lower rollback delays
Signing latency	Slower compared to verification	Acceptable as signing occurs only during checkpoint creation
Signature size	Larger than ECDSA/RSA	Increased storage overhead
Public-key size	Larger than classical schemes	Storage overhead is negligible over recovery efficiency
Deployment maturity	Standardized in FIPS 204	Better validation and proofing

In the proposed risk-aware rollback model, ML-DSA is used to sign checkpoint state hashes, verify checkpoint authenticity before rollback, prevent tampering with recovery state, and support trust-aware recovery decisions (Dinu, 2025). A checkpoint is created that includes information about the recovery state. A hash is assigned to this state. At the verifier level, the same hash is used for signing to ensure the authenticity of checkpoints for restoration. During rollback, integrity is checked first, and then the verified checkpoint is used in the recovery decision. The use of ML-DSA helps to ensure that the saved system state remains trustworthy even long after the system progresses, and even against future quantum-based adversaries (Olushola and Meenakshi, 2026).

#### 3.3.2 Modeling the post-quantum authenticated checkpoint

A checkpoint *W* created at time *t*, *W*_*t*_ is represented as:


$$\begin{array}{l} W_{t}=\left\{t,T_{t},M_{t},h_{t},{\text{Λ}}_{t}\right\} \end{array}$$ (1)

where

- *t* : timestamp of checkpoint creation
- *T*_*t*_ : checkpoint simulated service state of the system at time *t*
- *M*_*t*_ : metadata associated with the checkpoint
- *h*_*t*_ : cryptographic hash for ensuring integrity
- Λ_*t*_ : post-quantum digital signature for verifying authenticity using ML-DSA/CRYSTALS-Dilithium digital signature

The state of the system *T* is defined in this study as a checkpoint simulated system state, rather than the actual physical device states. *T*_*t*_ here is defined as the recoverable, runtime snapshot of the system state at time *t*. This definition considers recovery and rollback possibilities over the checkpoint-simulated system states using the state manager. Thus, the rollback mechanism operates at the checkpoint-simulated service-state layer by restoring a previously stored checkpoint, rather than reverting a physical device or the monitoring subsystem. The tuple *t, T*_*t*_, *M*_*t*_ refers to the payload of the checkpoint, which is serialized and then applied to the SHA-256 hash function to generate *h*_*t*_. i.e.,


$$\begin{array}{l} P_{t}=\left\{t,T_{t},M_{t}\right\} \end{array}$$ (2)

$$\begin{array}{l} h_{t}=H\left(P_{t}\right) \end{array}$$ (3)

where *H* refers to SHA-256 cryptographic hash function.

##### 3.3.2.1 Checkpoint verification using ML-DSA/CRYSTALS-Dilithium

In this study, an ML-DSA/CRYSTALS-Dilithium post-quantum signature verifier is implemented for verifying the checkpoints. The lattice-based signature design of CRYSTALS-Dilithium/NIST's module-lattice family of signature standards is utilized here. The trustworthiness of rollback checkpoints is validated using the ML-DSA/CRYSTALS-Dilithium verifier to enforce resilience against future quantum-capable adversaries, on which classic signature mechanisms like RSA and ECC fail. This validation phase is extremely significant as a compromised or vulnerable checkpoint selected for rollback could lead to added damage to the system. The signing and verification work in three steps as:

- Key generation: the (signing key, verifier key) pair is generated using a post-quantum key generation function KeyGen_PQ_() over a preset security parameter 1^λ^ as:

$$\begin{array}{l} \left(sg_{k},vf_{k}\right)\leftarrow \text{KeyGe}{\text{n}}_{\text{PQ}}\left(1^{\text{λ}}\right) \end{array}$$ (4)

- Checkpoint signing: the post-quantum digital signature Λ_*t*_ for the checkpoint is generated using the signing key *sg*_*k*_, over the hashed payload of the checkpoint *h*_*t*_, using the post-quantum signing function *Sign*_*PQ*_ as:

$$\begin{array}{l} {\text{Λ}}_{t}\leftarrow \text{Sig}{\text{n}}_{\text{PQ}}\left(sg_{k}\left(h_{t}\right)\right) \end{array}$$ (5)

- Checkpoint verification: the verification of the checkpoint is done using the generated verifier key *vf*_*k*_, as:

$$\begin{array}{l} \text{Verif}{\text{y}}_{PQ}\left(vf_{k}\left(h_{t},{\text{Λ}}_{t}\right)\right)\in \left\{0,1\right\} \end{array}$$ (6)

where 1 refers to a verified, authentic checkpoint and 0 refers to an unverified, potentially tampered or compromised checkpoint. Only those checkpoints that return 1 in verification will be considered for rollback. However, ML-DSA/CRYSTALS-Dilithium is used only to verify checkpoint authenticity and integrity and does not by itself ensure that a checkpoint is clinically valid, operationally safe, confidential, or free from malicious internal state. Therefore, the cryptographic layer is complemented by severity estimation, trust scoring, freshness-aware selection, and rollback policy logic to enhance the quality of recovery and rollback.

### 3.4 Checkpoint freshness and trust score calculation

Rolling back to a previous checkpoint is safe if and only if the selected checkpoint takes the system to a fresh and authentic state to resume. Here, freshness refers to how recent the selected checkpoint is to the current state of the system. Verification of the checkpoint using ML-DSA/CRYSTALS-Dilithium ensures the checkpoint has not been altered by an attacker. This combined assurance of freshness and authenticity makes the recovery process suitable for sensitive systems like healthcare IoT environments where both availability and integrity are critical.

The freshness of the checkpoint and its subsequent ranking is evaluated to select the most suitable checkpoint to roll back from the available multiple options. A more recent checkpoint might be easier to use to preserve the current operational state of the system, but an older checkpoint might be more trustworthy based on its better verification history. Freshness combined with ranking on trustworthiness of the selected checkpoint helps in the final selection of the most suitable checkpoint for rolling back on recovery.

#### 3.4.1 Defining freshness

The age of the checkpoint *C*_*i*_ can be calculated based on the current timestamp *t*_*current*_ and the saved timestamp *t*_*i*_ for *C*_*i*_, as in Equation 7:


$$\begin{array}{l} \delta_{C_{i}}=t_{current}-t_{i} \end{array}$$ (7)

To calculate the freshness score, the age of the selected checkpoint *C*_*i*_ is compared against that of the most recent checkpoint *C*_*current*_, as mentioned below in Equation 8:


$$\begin{array}{l} F_{C_{i}}=1-\frac{\delta_{C_{i}}}{\delta_{C_{current}}} \end{array}$$ (8)

resulting in a value between [0,1], where a value closer to 0 indicates an older checkpoint, whereas a value closer to 1 indicates a more recent checkpoint.

#### 3.4.2 Calculating the trust score for checkpoint

A ranking based on trust is calculated based on the classifier confidence score *c*(*v*) from tier1, freshness score *F*_*C*_*i*__, reconstruction error *e*(*v*), and the verification latency L__v_*i*_, as given below:


$${\text{R}}_{i}=w_{1}F_{C_{i}}+w_{2}c(v)+w_{3}(1-e(v))+w_{4}(1-{\text{L}}_{vi})$$ (9)

where *w*_1_, *w*_2_, *w*_3_, and *w*_4_ are weights with values 0.20, 0.35, 0.20, and 0.25, respectively. Higher freshness score, higher confidence score, lower reconstruction error, and lower verification latency thus contribute to a higher ranking score of the given checkpoint.

### 3.5 RL-inspired rollback decision

The level of rollback action is chosen based on a reinforcement learning-inspired selector, which computes the attack severity as a rule-based severity estimation policy. The severity is calculated as a bounded weight function of the combined anomaly score *s*(*v*) from tier1, normalized reconstruction error ê(*v*), and confidence score *c*(*v*). Equation 10 represents the same.


$$\begin{array}{l} \text{Γ}{\left(\text{v}\right)}_{i}=\min\left(1,w_{1}s{\left(v\right)}_{i}+w_{2}ê{\left(v\right)}_{i}+w_{3}c{\left(v\right)}_{i}\right) \end{array}$$ (10)

Based on the calculated severity score, the rollback decision is made as:


$$P(\text{Γ}(v))=\{\begin{array}{ll} \text{complete}\_\text{rollback}, & \text{Γ}(v)\geq 0.75\\ \text{partial}\_\text{rollback}, & 0.40\leq \text{Γ}(v)<0.75\\ \text{subtle}\_\text{recovery}, & \text{Γ}(v)<0.40 \end{array}$$ (11)

**Figure 4:**
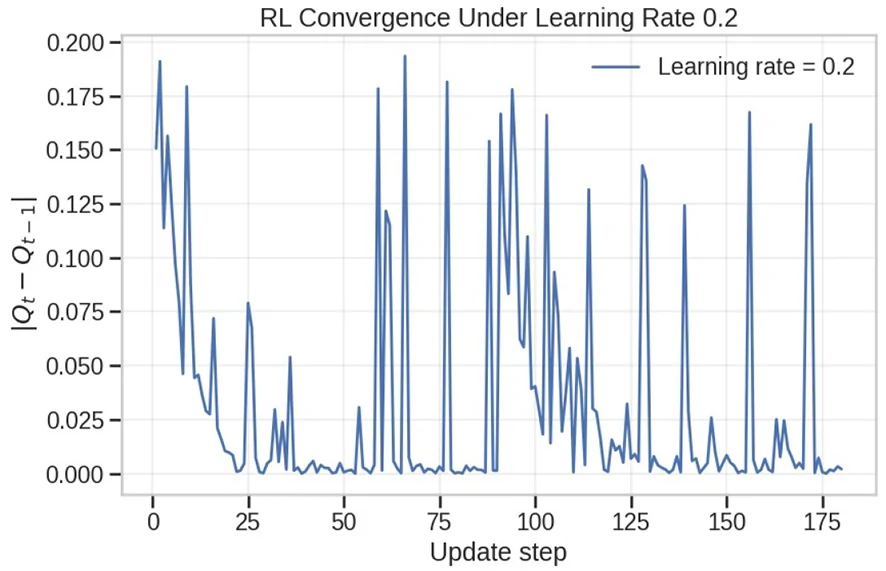
RL convergence with learning rate.

Subtle Recovery is chosen for cases where the attack severity is estimated to be very low, indicating that the system is not in any danger and only corrective action is required. Partial rollback is selected in cases of medium severity, implying there is a serious attack identified, and the system is restoring to a recent trusted state but avoids a full reset. Complete rollback is chosen when the attack severity is high, and it is important to initiate a complete reset of the system, moving it to a safe and authentic recovery point. Figure 4 represents the convergence of the RL-inspired Rollback Decision Classifier to the learning rate 0.2.

This rollback decision Equation 11 is then combined with the trust score derived in Equation 9 to initiate the final recovery decision. The checkpoint is chosen using the RL-inspired selector, as the one with the highest Q-point of all the candidate checkpoints. This is mathematically as in Equation 12:


$$\begin{array}{l} cp^{*}=\arg\underset{cp_{j}}{\max}Q\left(\text{Γ}{\left(v\right)}_{j},P_{j}\right) \end{array}$$ (12)

The standard reinforcement-learning idea of greedy action selection is used in this selection stage, where the checkpoint with the highest *Q*-value is chosen. The epsilon-greedy logic is also used in the implementation phase to ensure randomness, thus avoiding always selecting the same checkpoint. Timely update of the Q-table in this case is done as mentioned below in Equation 13:


$$\begin{array}{l} Q\left(\text{Γ}\left(v\right),P\right)\leftarrow Q\left(\text{Γ}\left(v\right),P\right)+\eta \left(r-Q\left(\text{Γ}\left(v\right),P\right)\right) \end{array}$$ (13)

where η is the learning rate and *r*, the reward value. Table 8 summarizes the reinforcement-learning-inspired rollback-decision used in the proposed system.

**Table 8:** Specification of the reinforcement-inspired checkpoint-selection policy used in the proposed framework.

Component	Notebook-based implementation
Environment	Policy receives attack severity and the set of verified checkpoints, in the self-healing pipeline
State space	Ranking the severity and freshness on a discrete state of the system based on attack severity and checkpoint freshness
Action space	Selection of one checkpoint timestamp from the set of available checkpoint candidates
Policy type	Lightweight reinforcement-learning-inspired Q-table policy selector
Exploration strategy	Epsilon-greedy action selection with ϵ = 0.15, allowing occasional random checkpoint selection
Q-table representation	Tabular *Q*(*s, a*) values indexed by discrete state and checkpoint timestamp
Update rule	Incremental *Q*-value update using learning rate *lr* = 0.2
Reward function	Reward derived from rollback quality, verification success, recovery efficiency, and severity penalty
Episode definition	Selection of a checkpoint is referred to as one decision episode
Interpretation	A heuristic, reinforcement-inspired policy selector

Self-Healing is enforced in the second tier of the proposed system. After detection and severity estimation of anomalies in the first tier, this second tier determines the level of recovery the system should undergo and also selects the best checkpoint to roll back using a post-quantum risk-aware scheme. The relevance of this approach in the healthcare IoT environment is its guarantee of service availability without compromising on security and integrity aspects. The multi-factor trust score calculation based on freshness, attack severity, reconstruction error, etc., and the recovery decision based on Q-points make the decision effective.

## 4 System threat model

In the proposed framework, an HIoT monitoring environment is considered where all endpoint medical or wearable devices generate network and service observations, which are then processed at the edge/gateway layers. Let $v_{i}\in {\mathbb{R}}^{d}$ denote the *i*-th preprocessed observation and let *V* ∈ ℝ^*N*×*d*^ denote the full feature matrix. The first tier estimates the attack probability *P*_*atk*_(*v*_*i*_), reconstruction-based deviation ê_*i*_, combined anomaly score *s*_*i*_, and severity score Γ_*i*_. The second tier performs recovery control by selecting a verified checkpoint and an appropriate rollback action.

A checkpoint created at time *t*_*i*_ is represented in Equation 1. The payload and hash are defined based on the checkpoint equation, as represented in Equations 2, 3, respectively. The checkpoint authentication module is implemented using an ML-DSA/CRYSTALS-Dilithium-based digital signature mechanism, utilizing the key generation, signing, and verification capacities (Equations 4–6). A checkpoint is eligible for rollback only when verification returns a 1. i.e., Verify_*PQ*_(*vf*_*k*_(*h*_*t*_, Λ_*t*_)) = 1}. Hence, the valid checkpoint can be represented as represented below in Equation 14


$$C_{valid}=\left\{W_{i}|{\text{Verify}}_{PQ}(vf_{k}(h_{t},\Lambda_{t}))=1\right\}.$$ (14)

### 4.1 Adversary capability

The adversary A is modeled as a probabilistic polynomial-time attacker with access to the network path and the checkpoint repository. The adversary may inject malicious traffic, replay earlier observations, cause abnormal service behavior, modify checkpoint metadata, replace checkpoint payloads, or attempt to force recovery to an unsafe checkpoint. The adversary may also replay an old checkpoint that was valid at an earlier time.

The adversary's actions are considered through four practical cases:

- Traffic manipulation: A modifies or generates observations *v*_*i*_ to reduce the anomaly score *s*_*i*_ or delay recovery.
- Checkpoint tampering: A modifies payload *P*_*t*_, *T*_*t*_, *M*_*t*_, or *h*_*t*_ while attempting to reuse Λ_*t*_.
- Checkpoint forgery: A attempts to create a new pair (h^*^, Λ^*^) that verifies under *pk* but was not signed using *sk*.
- Checkpoint replay: A reintroduces an old valid checkpoint *W*_*j*_ to influence recovery.

The adversary is not assumed to know the private signing key *sk*. The public key *pk* is assumed to be correctly bound to the checkpoint signing process. The model does not claim protection against physical device capture, malicious insiders, compromise of *sk*, or reversal of clinical actions. Rollback is limited to the checkpoint-simulated service state *T*_*t*_ at the monitoring or gateway layer.

### 4.2 Detection and recovery trigger

Equations 7–15 are used to represent the detection and recovery trigger in the proposed model. The first tier combines supervised attack evidence and reconstruction-based deviation:


$$\begin{array}{l} s_{i}=\alpha P_{atk}\left(v_{i}\right)+\left(1-\alpha \right){ê}_{i}, \end{array}$$ (15)

where α ∈ [0, 1]. The predicted label is


$$\hat{l}{\left(v\right)}_{i}=\{\begin{array}{ll} 1, & s_{i}\geq \tau_{s},\\ 0, & \text{otherwise}. \end{array}$$ (16)

The severity score is computed as


$$\begin{array}{l} \text{Γ}{\left(v\right)}_{i}=\min\left(1,0.45s{\left(v\right)}_{i}+0.35ê{\left(v\right)}_{i}+0.20c{\left(v\right)}_{i}\right) \end{array}$$ (17)

Recovery is triggered when the anomaly score or severity score exceeds the configured operational threshold.

### 4.3 Freshness-bounded candidate set

Signature verification proves authenticity, but it does not ensure by itself that a checkpoint is operationally recent. Equations 18–22 describes the calculation of freshness as mentioned in the proposed system. Therefore, rollback candidates are restricted by a freshness bound:


$$\begin{array}{l} C_{roll}\left(t_{cur}\right)=\left\{W_{i}\in C_{valid}|0\leq t_{cur}-t_{i}\leq T_{max}\right\}, \end{array}$$ (18)

where *t*_*cur*_ is the current time and *T*_*max*_ is the maximum allowed checkpoint age.

For ranking, let *W*_*j*_ denote a candidate checkpoint for the recovery event triggered by observation *x*_*i*_. The freshness score is computed based on Equation 8 as:


$$\begin{array}{l} F_{j}=\exp\left(-\frac{t_{cur}-t_{j}}{T_{f}}\right), \end{array}$$ (19)

where *T*_*f*_ is the freshness horizon. Let *L*_*j*_ denote the measured ML-DSA/CRYSTALS-Dilithium verification latency and ${\hat{L}}_{j}\in \left[0,1\right]$ its normalized value. If the rollback distance or expected data loss is estimated for a checkpoint, denote it by *D*_*j*_, with the normalized value ${\hat{D}}_{j}$. For each $W_{j}\in C_{roll}\left(t_{cur}\right)$, the ranking score is


$$\begin{array}{l} R_{j}=w_{1}F_{j}+w_{2}\left(1-{\hat{L}}_{j}\right)+w_{3}H_{j}+w_{4}\left(1-{\hat{D}}_{j}\right), \end{array}$$ (20)

where *H*_*j*_ is the reliability of the historical checkpoint, if maintained. The weights satisfy


$$\begin{array}{l} \overset{4}{\underset{k=1}{\sum }}w_{k}=1,\;w_{k}\geq 0. \end{array}$$ (21)

If *H*_*j*_ or ${\hat{D}}_{j}$ is not maintained, the corresponding term is removed and the remaining weights are renormalized. The anomaly severity Γ_*i*_ determines the level of recovery action, while *R*_*j*_ ranks candidate checkpoints. This separation avoids using event-level severity as though it were a checkpoint-specific property. The selected checkpoint is


$$\begin{array}{l} cp^{*}=\arg\underset{W_{j}\in C_{roll}\left(t_{cur}\right)}{\max}R_{j}. \end{array}$$ (22)

If $C_{roll}\left(t_{cur}\right)=\emptyset$, the system should enter a safe degraded mode and raise an administrative alert rather than rolling back to an unverified or stale state.

### 4.4 Rollback action

Recovery is proportional to the estimated severity. The action set is represented below (Equation 23) as:


$$\begin{array}{l} A=\left\{\text{subtle recovery},\text{partial rollback},\text{complete rollback}\right\}. \end{array}$$ (23)

A compact severity-based action mapping is provided in Equation 11. If a learning-based policy is used, the decision state may be written as shown in Equation 24


$$\begin{array}{l} S_{i}=\left\{{\text{Γ}}_{i},F_{i},{\hat{L}}_{i},R_{i}\right\}, \end{array}$$ (24)

and the selected action as in Equation 25


$$\begin{array}{l} a_{i}^{*}=\arg\underset{a\in A}{\max}Q\left(S_{i},a\right). \end{array}$$ (25)

### 4.5 Security objectives

The model is designed around four objectives.

- Detection sensitivity: malicious observations should produce sufficient anomaly evidence through *s*_*i*_ and Γ_*i*_.
- Checkpoint authenticity: only checkpoints satisfying Verify_*PQ*_(*vf*_*k*_(*h*_*t*_, Λ_*t*_)) = 1 should be eligible for recovery.
- Replay and staleness control: previously valid but stale checkpoints should be excluded by *T*_*max*_ and down-ranked by *F*_*i*_.
- Risk-aware recovery: rollback should be proportional to severity and should prefer recent, verified, and low-latency checkpoints.

** Claim 1 (Resistance to checkpoint tampering and forgery)**. Assume that the implemented ML-DSA/CRYSTALS-Dilithium signature scheme is existentially unforgeable under chosen-message attacks and that *H*(·) is collision-resistant. For any adversary A that does not know *s*_*k*_, the probability of producing a forged or tampered checkpoint W^*^ such that $mathrm{Verify}_{PQ}{\left(vf_{k}\left(h_{t},{\text{Λ}}_{t}\right)\right)}^{*}=1$ is negligible:


$$\begin{array}{l} \mathrm{Pr}\left[mathrm{Verify}_{PQ}{\left(vf_{k}\left(h_{t},{\text{Λ}}_{t}\right)\right)}^{*}=1\right]\leq \text{Ad}{\text{v}}_{\text{ML}\text{-DSA}}^{EUF\text{-}CMA}\left(A\right)\\ +Adv_{H}^{coll}\left(A\right). \end{array}$$ (26)

Equation 26 shows the mathematical representation of the claim.

*Proof*. If A modifies the checkpoint payload but reuses an old signature, then the hash value changes unless a collision in *H*(·) is found. If A creates a new valid signature for an unsigned hash, then it breaks the unforgeability of the ML-DSA/CRYSTALS-Dilithium signature scheme. Under the stated assumptions, both events have negligible probability.

** Claim 2 (Exclusion of stale replayed checkpoints)**. A previously valid checkpoint *W*_*j*_ can enter the rollback candidate set only if it satisfies the freshness as represented together by Equations 27, 28:


$$\begin{array}{l} 0\leq t_{cur}-t_{j}\leq T_{max}. \end{array}$$ (27)

For stale checkpoints where *t*_*cur*_−*t*_*j*_>*T*_*max*_,


$$\begin{array}{l} \mathrm{Pr}\left[W_{j}\in C_{roll}\left(t_{cur}\right)\right]=0. \end{array}$$ (28)

*Proof*. By definition, $C_{roll}\left(t_{cur}\right)$ contains only checkpoints from $C_{valid}$ whose age is at most *T*_*max*_. Therefore, even if *W*_*j*_ has a valid signature, it is excluded when it violates the freshness bound.

** Claim 3 (Verified rollback eligibility)**. The selected rollback checkpoint cp^*^ is always verified and freshness-bounded:


$$\begin{array}{l} cp^{*}\in C_{roll}\left(t_{cur}\right)\Rightarrow b_{cp^{*}}=1\;\text{and }t_{cur}-t_{cp^{*}}\leq T_{max}. \end{array}$$ (29)

*Proof*. The selection rule maximizes *R*_*i*_ only over $W_{i}\in C_{roll}\left(t_{cur}\right)$. Since $C_{roll}\left(t_{cur}\right)\subseteq C_{valid}$, every selectable checkpoint (as shown in Equation 29) must satisfy *b*_*i*_ = 1. The freshness bound also follows directly from the definition of $C_{roll}\left(t_{cur}\right)$.

These claims establish authenticity, freshness-bounded eligibility, and risk-aware selection for the proposed self-healing recovery layer. They do not claim confidentiality of checkpoint contents, protection after signing-key compromise, or correctness of clinical actions.

## 5 Results and discussions

The following section describes in detail the results and findings of the comparative analysis of the proposed system with other baseline models.

### 5.1 Experimental setup and implementation details

All experiments were carried out using the Google Colab platform, which provides a hosted Jupyter notebook environment with a virtualized CPU and sufficient main memory to process both datasets. The implementation of the proposed two-tier detection and recovery, self-healing model for healthcare IoT using post-quantum verification, was done in Python 3.12.12 [Python Software Foundation (PSF), python.org.], and all reported results correspond to this cloud-based CPU runtime (no GPU-specific optimizations were used). The same configuration was maintained across all runs for a fair comparison with the baseline methods. In all experiments, the reported metrics (attack probability, anomaly score, trust score, uptime, recovery time, severity analysis, and rollback quality) are computed deterministically over the complete test samples of both the WUSTL EHMS 2020 and CIC IoMT 2024 datasets for each method. The baselines are chosen based on detection, recovery, and rollback parameters: Detection only, Recovery only, and Rollback only. The baseline implementations are run with fixed configurations, yielding identical results up to negligible numerical differences over the same environmental settings. For this reason, we report single aggregate values per method and do not attach error bars or confidence intervals around the curves in the figures and tables. Table 9 consolidates the details of the experimental environment.

**Table 9:** Experimental environment and dataset characteristics for the evaluation of the proposed hybrid framework.

Execution environment	
Platform	Google Colab
Runtime	Linux 6.6.105+ (x86_64, glibc 2.35); Python 3.12.12
15.6-8.1,-13.5243ptCore libraries	NumPy 2.0.2; scikit-learn 1.6.1; pandas 2.2.2; Matplotlib 3.10.0; Dilithium_py
Dataset details	
15.6-8.1,-1.3243ptModel	WUSTL EHMS 2020: 4.4 MB CSV file
Dataset split and training	
Type	Size
Training data	70% of the dataset
Validation	20% of the training data
Testing	30% of the dataset
15.6-8.1,-1.3243ptModel	CIC IoMT 2024: 268 MB CSV file
Dataset split and training	
Type	Size
Training Data	70% of the dataset
Validation	20% of the training data
Testing	30% of the dataset

The ML-DSA/CRYSTALS-Dilithium operations were implemented using oqs-python (Open Quantum Safe bindings) in the Google Colab runtime. The cryptographic benchmark was conducted on *N* = 1, 620 checkpoint records. For each record, the serialized checkpoint payload was hashed, signed, and subsequently verified. Key generation, signing, and verification were measured separately using repeated high-resolution wall-clock timing. The mean latencies were 1.84 ± 0.13 ms per key pair for key generation, 0.96 ± 0.07 ms per record for signing, and 0.625 ± 0.042 ms per record for verification. Verifier throughput is computed as *N* divided by the total wall-clock verification time (seconds) over the full batch. The measured verification latency corresponds to a throughput of approximately 1,600 records per second and a cumulative verification time of about 1.013 s. These measurements represent software-level cryptographic processing and do not include the physical recovery time of deployed healthcare IoT devices. The benchmark results are summarized in Table 10.

**Table 10:** ML-DSA/CRYSTALS-Dilithium performance using oqs-python in the Google Colab CPU evaluation environment.

Metric	Measured value
Key-generation latency (ms/key pair)	1.84 ± 0.13
Signing latency (ms/record)	0.96 ± 0.07
Verification latency (ms/record)	0.625 ± 0.042
Verifier throughput (records/s)	≈1, 600
Number of verified records, *N*	1, 620
Cumulative verification time for *N* records (s)	≈1.013

Values are reported as mean ± sample standard deviation. Verifier throughput was calculated from the cumulative wall-clock verification time. The cumulative verification time is approximately 1, 620 × 0.625 ms = 1.013 s.

### 5.2 Metrics for comparison

The metrics chosen for evaluating the performance of the proposed model with standard baselines are detailed below.

#### 5.2.1 Attack probability

The probability of a given sample being malicious is estimated in this metric. A higher value represents higher confidence of the model that the event is an attack. It contributes to the overall performance of the model as it helps to distinguish attack traffic from normal traffic, and has significant importance in the rollback decisions.

#### 5.2.2 Anomaly score

This metric computes the deviation of the given sample's behavior from the expected normal behavior. In this research, this metric is used to improve the robustness of the detection phase, as the anomaly score value helps provide better accuracy in classifying the sample.

#### 5.2.3 Trust score

This multi-factor-based metric represents the overall confidence in the selected checkpoint for rollback upon the occurrence of an attack event. This value is computed over classifier confidence, severity, checkpoint freshness, and verification latency. Higher trust scores suggest a safer recovery choice.

#### 5.2.4 Uptime

This resilience metric directly analyses the availability of the system services. It evaluates the percentage of time the system can remain operational and provide the assured services. Higher values of uptime mean better service continuity during or after attacks.

#### 5.2.5 Recovery time

Time needed to restore the normal operations of the system after detection of an attack or after a system failure is evaluated in this metric. Lower values of recovery time imply faster return to normal behavior.

#### 5.2.6 Severity analysis

Severity Analysis refers to the impact of a potential attack or unusual event. This helps to understand the seriousness of the detected unusual event, potentially an attack, which also helps in efficient rollback decision. The higher the severity estimated, the more aggressive the rollback should also be. Lower levels of severity will lead to micro recovery without disrupting the system operations.

#### 5.2.7 Rollback quality

This is a combinatorial metric that reflects trust, verification success, severity, and speed. It evaluates the effectiveness of the recovery action carried out, thus highlighting the overall system performance.

#### 5.2.8 F1-score, precision, recall and false positive rate

These standard metrics are used to evaluate the performance of the model, over the characteristic behaviors of the samples and prompt identification of them into malicious and benign ones by the model under examination.

The metrics listed above are chosen to compare the performance of the proposed model against the baselines, as they help evaluate detection, recovery, rollback, and overall system effectiveness. Higher values of anomaly and attack probability scores help in efficient detection of attack traffic, whereas the severity score estimates the impact of a potential attack, and uptime, trust score, and rollback quality metrics help in evaluating the effectiveness of recovery.

### 5.3 Comparative performance evaluation with baseline models

The proposed model was compared with other standard baseline models for detection, rollback, and recovery over both WUSTL EHMS 2020 and CIC IoMT 2024 datasets. The standard baselines were chosen for this comparison as Detection Only (MLPOnlyDetector, AutoEncodeOnlyDetector, FusionNoRollBackDetector), Rollback(NoPolicyRollback, RandomCheckPointRollback, LatestCheckpointRollback, StaticRollbackBaseline, StaticThresholdRollback). Table 11 consolidates the baseline models chosen for the comparative analysis.

**Table 11:** Baseline models for comparative analysis.

Baseline model	Model type	Description
**MLPOnlyDetector**, (Sahitya et al., 2024; Upender et al., 2025)	Detection only	Uses only the supervised MLP classifier output as an attack score along with a preset threshold, without using the autoencoder score
**AutoEncodeOnlyDetector**, (Neloy and Turgeon, 2024; Oleiwi and Hadi, 2026)	Detection only	Uses only reconstruction error from the autoencoder and a threshold, omits the MLP classifier output
**FusionNoRollBackDetector**, (Zhang, 2025; Zhang et al., 2025)	Detection only	Combines MLP classifier output and autoencoder anomaly score, without initiating a rollback
**NoPolicyRollback**, (Mezzina et al., 2025; de Souza and de Freitas, 2025)	Rollback	No learned policy for rollback, always rolls back to the newest available verified checkpoint
**RandomCheckPointRollback**, (Aftab et al., 2026; Doku and Dinda, 2025)	Rollback	The checkpoint to rollback is selected uniformly at random
**LatestCheckpointRollback**, (Ltaief et al., 2026; Jing et al., 2026)	Rollback	The newest verified checkpoint is chosen for rolling back
**StaticRollbackBaseline**, (Pittorino and Roveri, 2026; Mehrban et al., 2025)	Rollback	Uses a fixed non-adaptive rollback rule to select the rollback checkpoint
**StaticThresholdRollback**, (Mwangi et al., 2025)	Rollback	The decision to roll back is taken only when the severity score exceeds a preset threshold

The proposed post-quantum verified self-healing model performs well over the baselines. The proposed model also considers the verification latency incurred in the process of checkpoint verification using the ML-DSA/CRYSTALS-Dilithium verifier. The results of comparison over the metrics discussed above for both datasets are detailed below in Figures 5–15.

**Figure 5:**
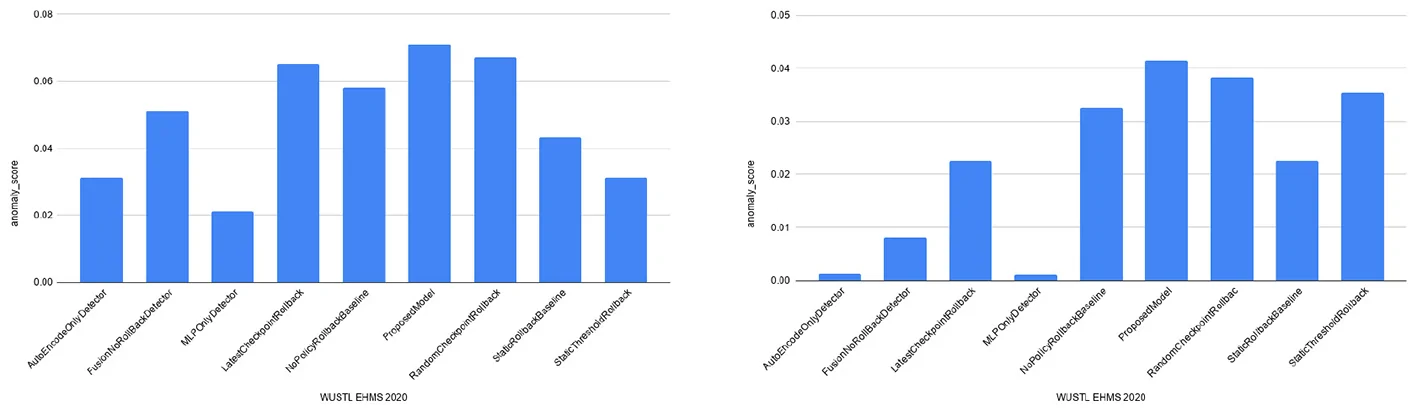
Anomaly score calculation. **(A)** Over CIC IoMT 2024 dataset. **(B)** Over WUSTL EHMS 2020 dataset.

**Figure 6:**
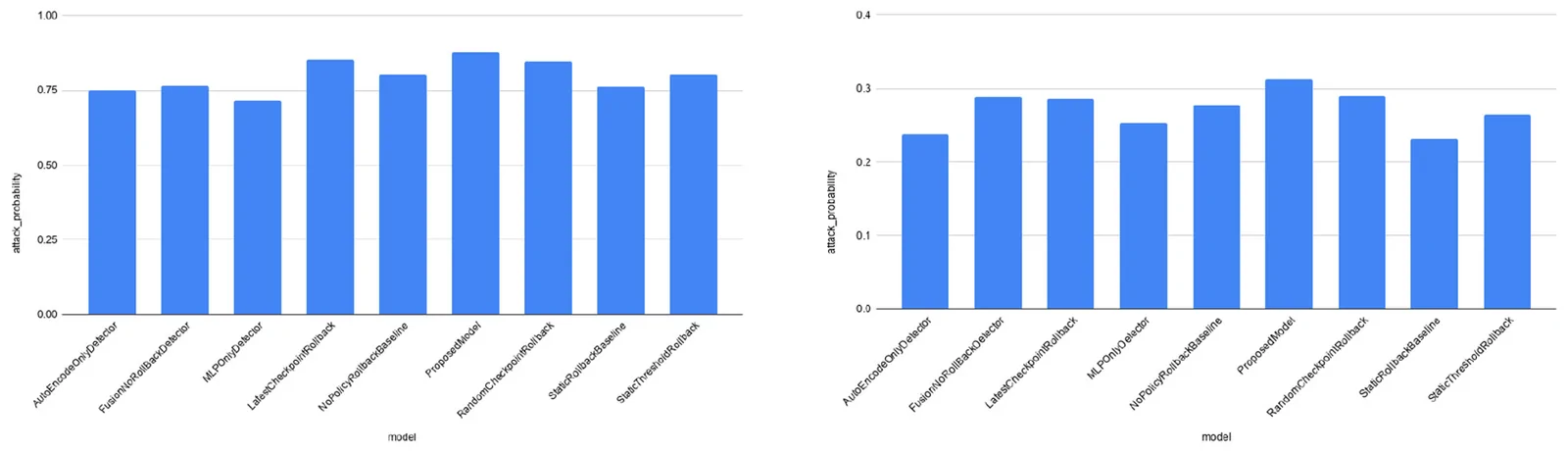
Attack probability calculation. **(A)** Over CIC IoMT 2024 dataset. **(B)** Over WUSTL EHMS 2020 dataset.

**Figure 7:**
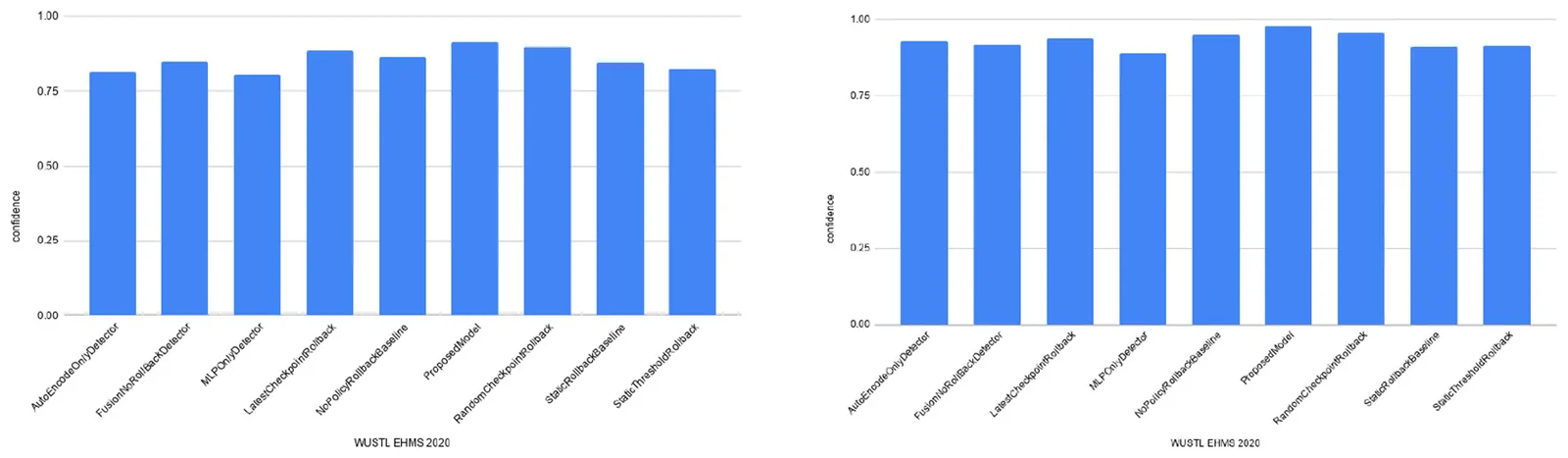
Confidence score calculation. **(A)** Over CIC IoMT 2024 dataset. **(B)** Over WUSTL EHMS 2020 dataset.

**Figure 8:**
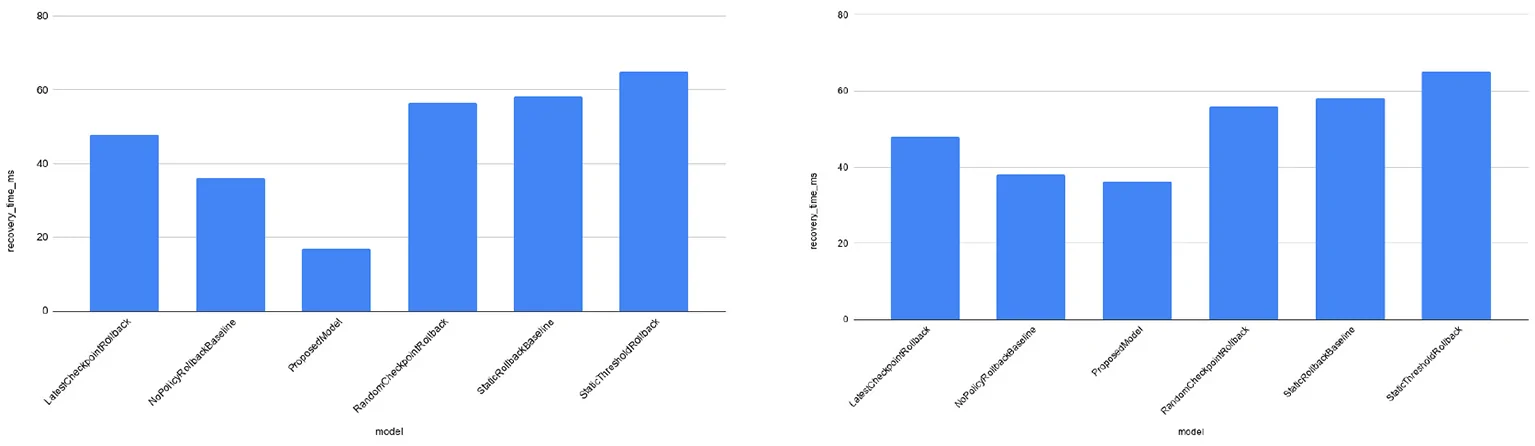
Recovery time in nanoseconds. **(A)** Over CIC IoMT 2024 dataset. **(B)** Over WUSTL EHMS 2020 dataset.

**Figure 9:**
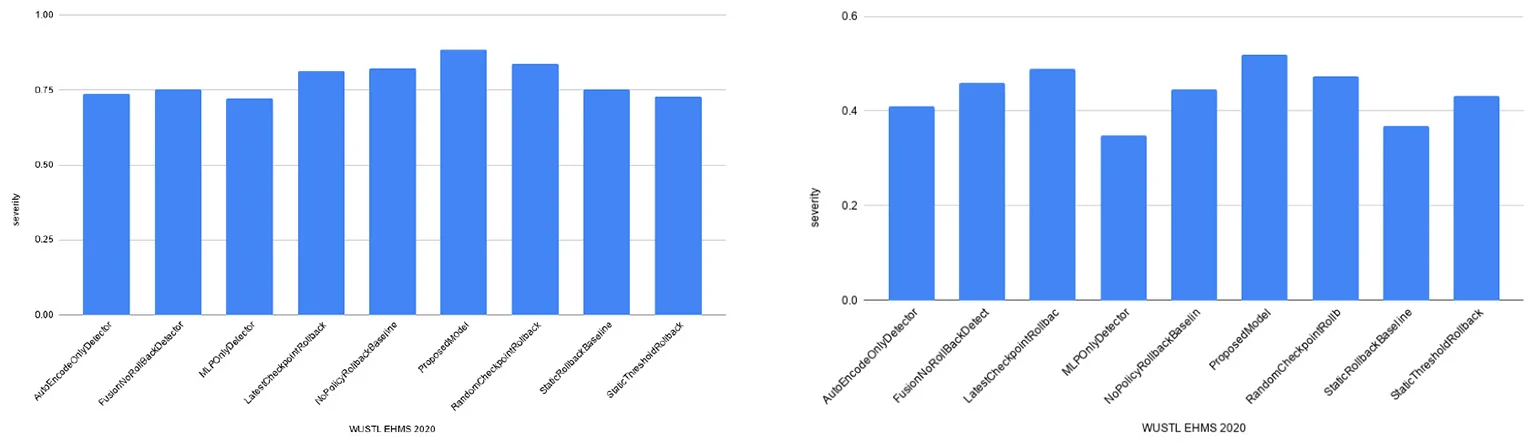
Severity estimation. **(A)** Over CIC IoMT 2024 dataset. **(B)** Over WUSTL EHMS 2020 dataset.

**Figure 10:**
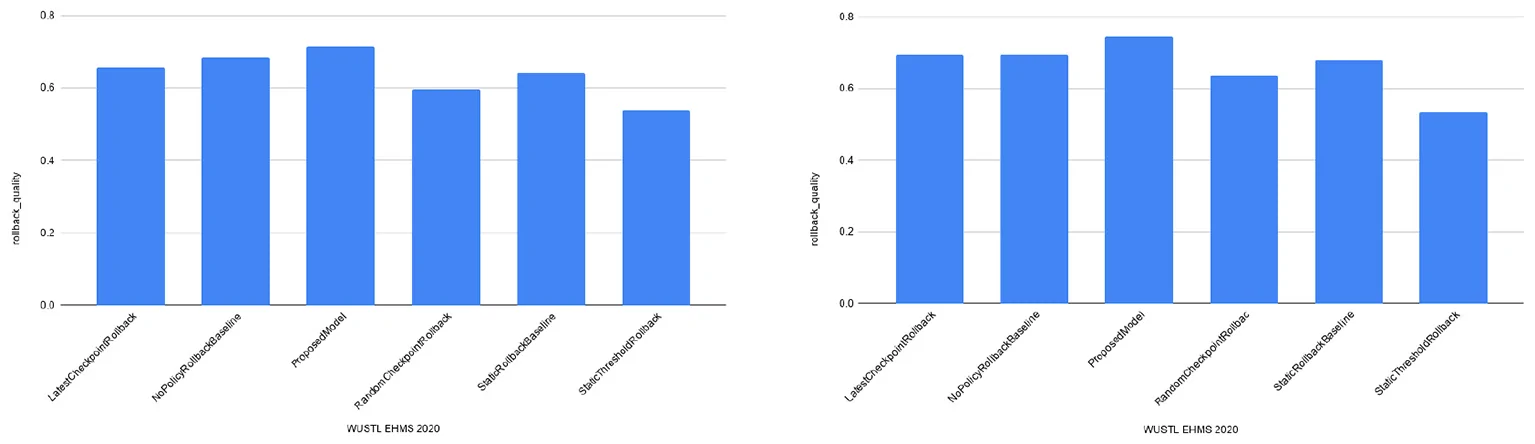
Quality of rollback decision. **(A)** Over CIC IoMT 2024 dataset. **(B)** Over WUSTL EHMS 2020 dataset.

**Figure 11:**
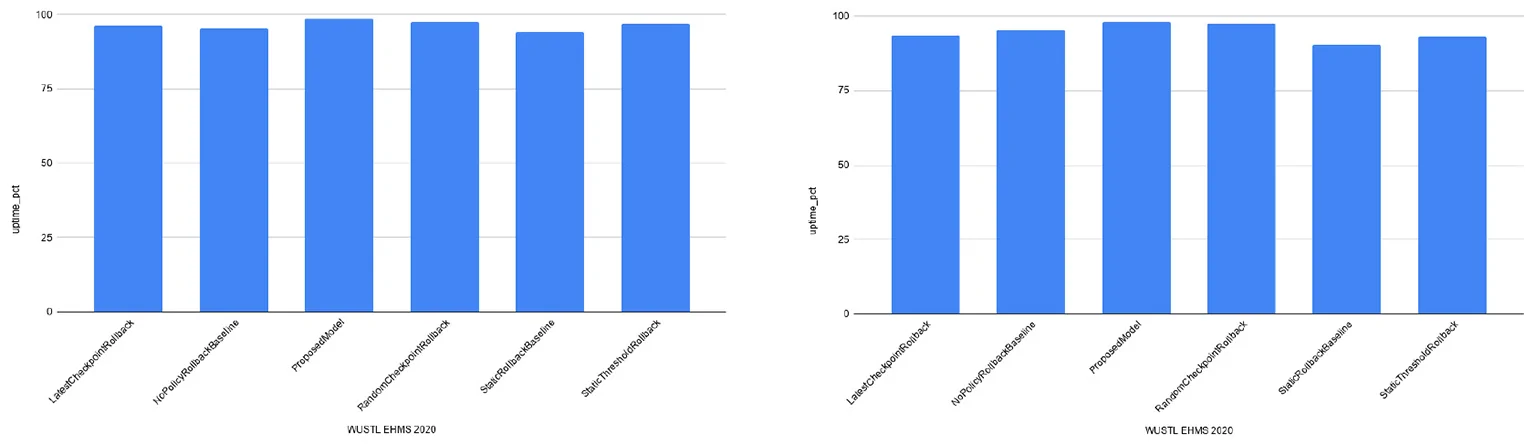
Uptime estimation. **(A)** Over CIC IoMT 2024 dataset. **(B)** Over WUSTL EHMS 2020 dataset.

**Figure 12:**
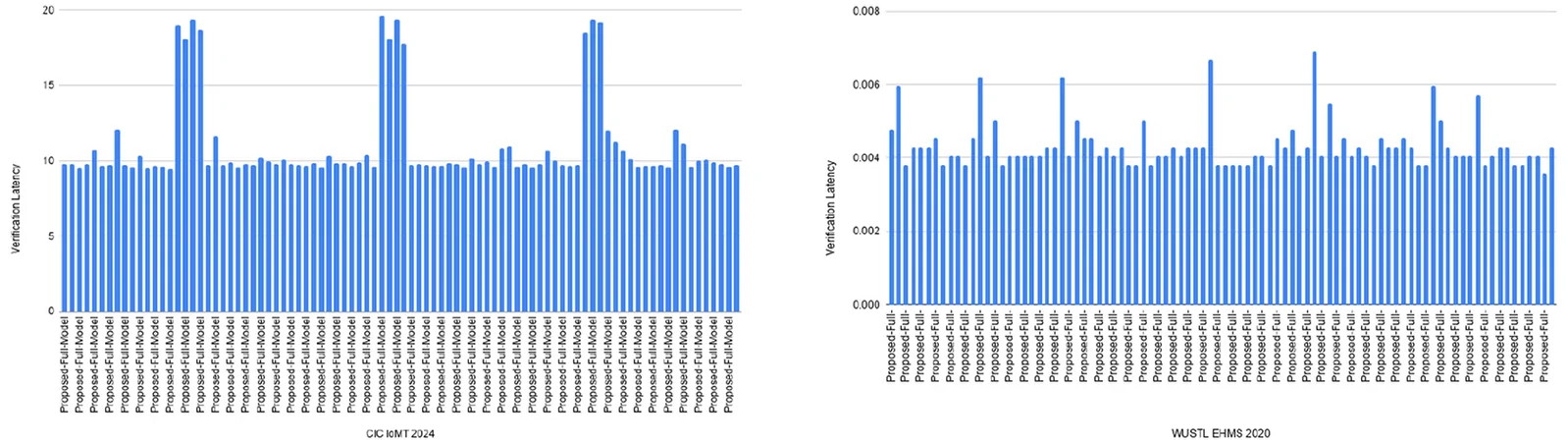
Verification latency of proposed model. **(A)** Over CIC IoMT 2024 dataset. **(B)** Over WUSTL EHMS 2020 dataset.

**Figure 13:**
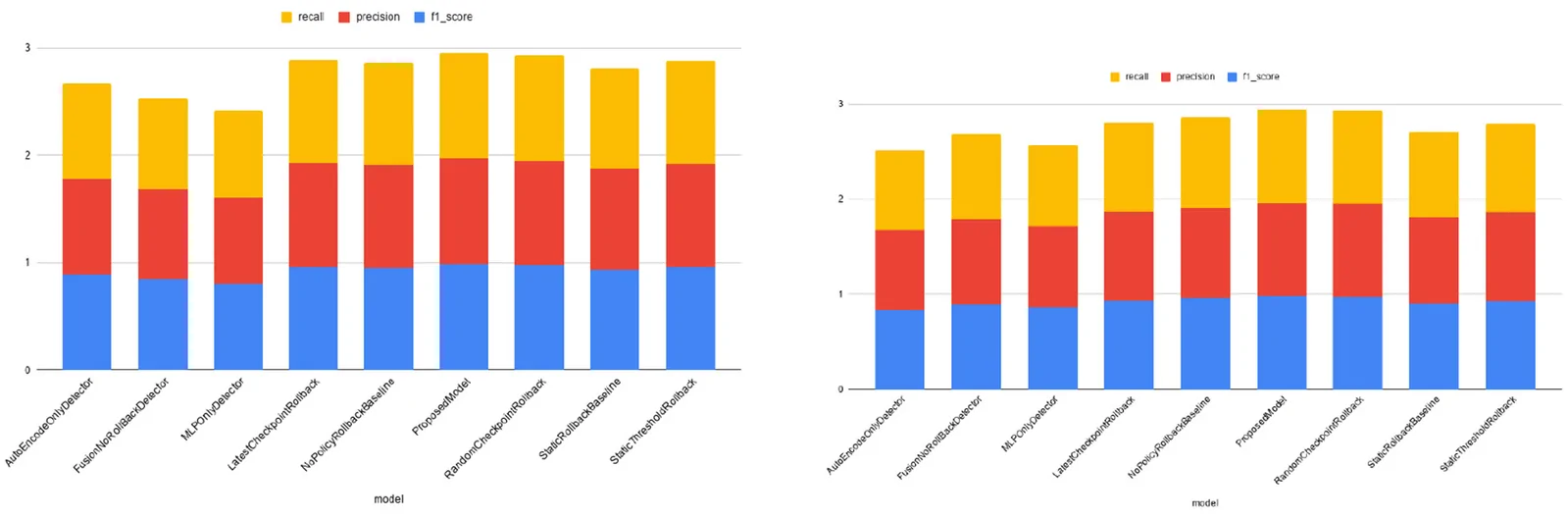
F1-score, recall and precision. **(A)** Over CIC IoMT 2024 dataset. **(B)** Over WUSTL EHMS 2020 dataset.

**Figure 14:**
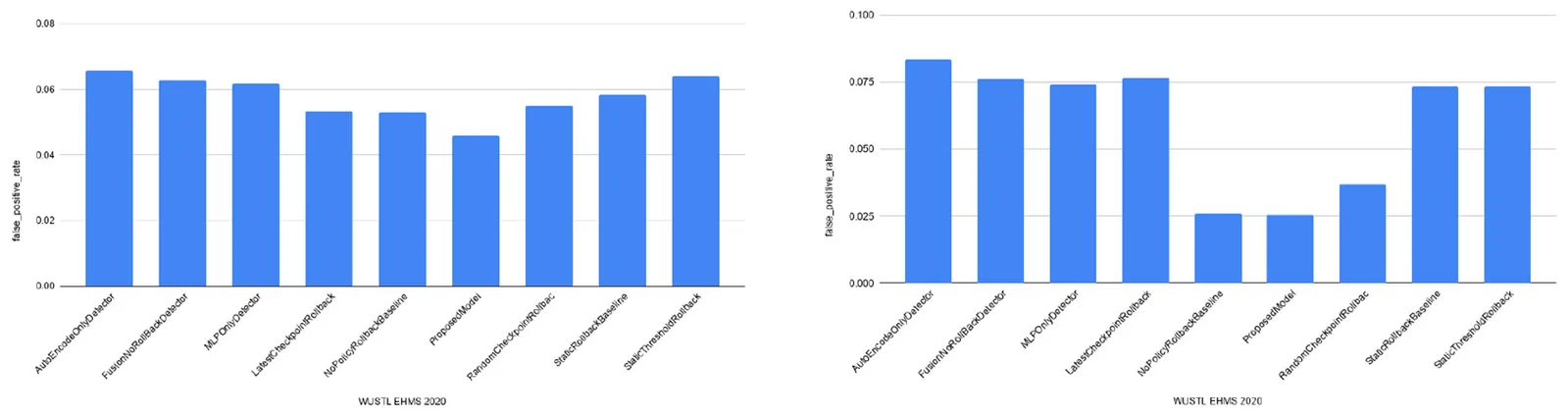
False positive rate. **(A)** Over CIC IoMT 2024 dataset. **(B)** Over WUSTL EHMS 2020 dataset.

**Figure 15:**
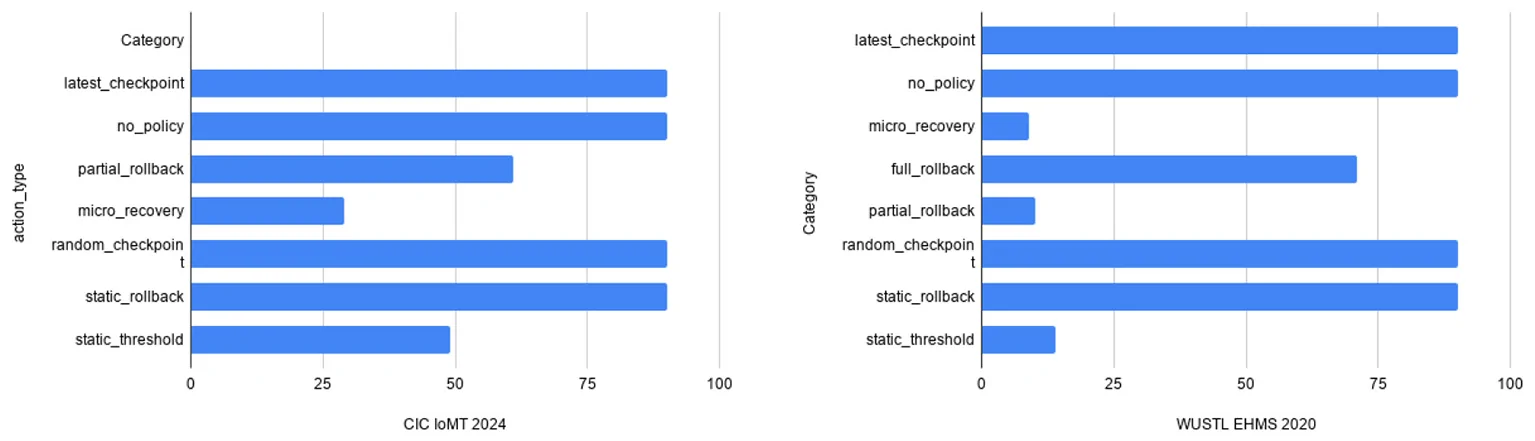
Rollback choices selected by models cumulative. **(A)** Over CIC IoMT 2024 dataset. **(B)** Over WUSTL EHMS 2020 dataset.

Based on the measured values of accuracy, precision, recall, and F1-score, the proposed model is analyzed over the ROC-AUC, PR-AUC, and MCC. Table 12 consolidates the findings.

**Table 12:** Performance of baselines across datasets using ROC-AUC, PR-AUC, and MCC.

Dataset	Models	ROC-AUC	PR-AUC	MCC
WUSTL EHMS 2020	Proposed Full-Model	0.978 ± 0.12	0.981 ± 0.15	0.954 ± 0.12
	MLPOnlyDetector	0.862 ± 0.24	0.856 ± 0.28	0.628 ± 0.23
	AutoEncodeOnlyDetector	0.842 ± 0.18	0.876 ± 0.14	0.613 ± 0.17
	NoPolicyRollback	0.959 ± 0.15	0.955 ± 0.12	0.905 ± 0.13
	FusionNoRollbackDetector	0.901 ± 0.21	0.891 ± 0.19	0.788 ± 0.24
	LatestCheckpointRollback	0.942 ± 0.11	0.936 ± 0.15	0.867 ± 0.23
	StaticThresholdRollback	0.8936 ± 0.23	0.930 ± 0.27	0.821 ± 0.16
	RandomCheckpointRollback	0.979 ± 0.13	0.976 ± 0.11	0.821 ± 0.12
CICIoMT24	Proposed Full-Model	0.985 ± 0.13	0.981 ± 0.14	0.971 ± 0.13
	MLPOnlyDetector	0.804 ± 0.23	0.801 ± 0.25	0.607 ± 0.22
	AutoEncodeOnlyDetector	0.889 ± 0.18	0.839 ± 0.14	0.758 ± 0.17
	NoPolicyRollback	0.9529 ± 0.15	0.9514 ± 0.12	0.9058 ± 0.13
	FusionNoRollbackDetector	0.843 ± 0.21	0.834 ± 0.19	0.685 ± 0.24
	LatestCheckpointRollback	0.963 ± 0.11	0.961 ± 0.15	0.926 ± 0.23
	StaticThresholdRollback	0.956 ± 0.23	0.941 ± 0.25	0.923 ± 0.18
	RandomCheckpointRollback	0.975 ± 0.12	0.972 ± 0.11	0.952 ± 0.14

The comparative analysis with standard baselines for detection and rollback showed that the proposed two-tier model with deep anomaly detection, post-quantum verification, and risk-aware rollback performed better than the existing standard baselines in both detection and recovery aspects. The higher average values of attack probability, anomaly score, severity, and confidence score proved that the proposed model promptly identifies and detects any potential attack sample from the whole traffic under analysis, along with a quantitative measure of its impact. Higher values of average trust score and rollback quality show that the proposed model enforces better recovery measures, with the checkpoint selected based on post-quantum verification. Furthermore, higher values of F1 score, precision, recall, and uptime, as well as lower values for average verification latency, false positive rate, and recovery time, proved the efficiency of the proposed model without compromising both detection and recovery aspects.

### 5.4 Ablation analysis with baseline models

The proposed model achieved strong overall performance across both healthcare IoT datasets, with average values of 59.23% attack probability, 94.00% confidence, 5.61% anomaly score, 69.50% severity, 26.61 ns recovery time, 75.86% trust score, 98.37% uptime, 72.93% rollback quality, 0.58ms verification latency, 98.37% F1-score, 98.28% recall, 98.4% precision, and 0.45% false positive rate, on random seed 42. An ablation study was performed to recheck the system's performance by removing one component at a time to analyze the contribution of each component to overall performance. The results of this study are tabulated in Tables 13, 14.

**Table 13:** Ablation analysis of the proposed framework with detection metrics.

Variant	Accuracy	Precision	Recall	F1-score	FPR	Inference
Proposed Full-Model	0.9823	0.9849	0.9828	0.9837	0.045	Balanced performance with efficient detection, classification, analysis, and rollback decision
Without MLP classifier	0.8314	0.8128	0.9167	0.9043	0.321	Lower values for accuracy and precision as the classification decision is not accurate
Without autoencoder	0.9427	0.9343	0.8146	0.7989	0.351	Anomaly sensitivity is lowered due to unavailability of class-based reconstruction anomaly evidence
Without fusion	0.9334	0.9127	0.9284	0.8143	0.389	Higher rates for FPR or low F1 score, owing to only one signal input
Without post-quantum verification	0.9818	0.9837	0.9824	0.9803	0.047	No cryptographic verification faster rollback, but compromised on security
Without trust-score calculation	0.9823	0.9843	0.9825	0.9831	0.045	Faster computation but compromises multi-factor-based trust score calculation for better authenticity, as the risk-aware property is eliminated
Without RL-inspired selector	0.9815	0.9827	0.9826	0.9812	0.048	The adaptive property of the system in choosing the rollback level is eliminated; the decision is static

**Table 14:** Ablation analysis of the proposed framework with recovery metrics.

Variant	Recovery time	Verification latency	Trust score	Uptime	Rollback quality	Inference
Proposed Full-Model	26.61 ns	0.58 ms	0.7586	0.9837	0.7293	Balanced decision making and accurate rollback decision based on exact classification of attack
Without MLP classifier	29.12 ns	0.63 ms	0.7845	0.9724	0.6872	Rollback decisions are unstable because of poor classifications
Without autoencoder	29.21 ns	0.62 ms	0.7045	0.9645	0.7132	Recovery and rollback decisions are static as the anomaly classification is minimal
Without fusion	28.45 ns	0.65 ms	0.7358	0.9351	0.7154	Rollback and recovery decisions are made based on single classifier output
Without post-quantum verification	21.27 ns	0.68 ms	0.6824	0.9718	0.6587	Faster recovery time as post-quantum verification is eliminated, but low values for rollback quality and trust
Without trust-score calculation	25.15 ns	0.60 ms	0.000	0.9423	0.6515	Lower uptime and rollback quality; risk-aware property of checkpoint is eliminated
Without RL-inspired selector	28.16 ns	0.61 ms	0.7315	0.8542	0.6875	Rollback decisions are unstable, mostly static

### 5.5 Comparison with state-of-the-art intrusion detection models

The performance of the proposed model is also compared against a few state-of-the-art intrusion detection models, proposed for IoT systems—interpretable CNN-BiLSTM permutation importance framework (Al-Shibly et al., 2026), AutoEncoder based feature learning (Hasan et al., 2025), temporal attention CNN (Ghosh et al., 2025), Federated Learning and Resource Aware GNN (Ma et al., 2026), over the metrics of accuracy, precision, recall, f1-score and false positive rate. Table 15 consolidates the results of the comparative analysis. The results show that the proposed model achieves enhanced performance compared to existing state-of-the-art models in terms of better accuracy, recall, precision, and F1-score, and a reduced false positive rate.

**Table 15:** Comparison with recent intrusion detection systems.

Study	Method	Accuracy	Precision	Recall	F1-score	FPR
Proposed framework	MLP+ autoencoder +risk-aware rollback	0.9823	0.9843	0.9789	0.9819	0.0113
Enhanced intrusion detection in IIoT networks	Autoencoder-based lightweight IDS	0.9804	0.9775	0.9789	0.9801	0.0213
CNN-BiLSTM permutation framework	CNN-BiLSTM feature selection	0.9718	0.9734	0.9723	0.9724	0.0287
Federated Learning for IoT	Federated Learning and Resource Aware GNN	0.9473	0.9451	0.9379	0.9481	0.0687

### 5.6 Statistical aggregation results

The performance of the proposed model is also statistically analyzed over 10 different runs with distinct random seed values to prove the performance quality. The runs were carried out on both datasets, and the results are consolidated in Tables 16, 17. The best performance was found with seed value 42 for both datasets. Table 18 consolidates the analytical observations of the statistical runs.

**Table 16:** Statistical aggregation of results across 10 runs–WUSTL dataset.

Run	Seed	Accuracy	Precision	Recall	F1-score	FPR
1	42	0.9823	0.9843	0.9789	0.9819	0.0163
2	68	0.9518	0.9562	0.9467	0.9514	0.0161
3	11	0.9687	0.9709	0.9654	0.9681	0.0139
4	97	0.9345	0.9398	0.9281	0.9339	0.0207
5	23	0.9604	0.9631	0.9578	0.9604	0.0150
6	54	0.9731	0.9756	0.9698	0.9727	0.0124
7	76	0.9459	0.9496	0.9412	0.9454	0.0189
8	19	0.9655	0.9680	0.9621	0.9650	0.0144
9	88	0.9572	0.9607	0.9528	0.9567	0.0168
10	31	0.9798	0.9817	0.9763	0.9790	0.0119

**Table 17:** Statistical aggregation of results across 10 runs–CICIoMT24 dataset.

Run	Seed	Accuracy	Precision	Recall	F1-score	FPR
1	42	0.9843	0.9832	0.9809	0.9854	0.0146
2	68	0.9731	0.9756	0.9698	0.9727	0.0149
3	11	0.9798	0.9817	0.9763	0.9790	0.0139
4	97	0.9234	0.9236	0.9265	0.9327	0.0212
5	23	0.9655	0.9680	0.9621	0.9650	0.0144
6	54	0.9518	0.9562	0.9467	0.9514	0.0167
7	76	0.9419	0.9468	0.9407	0.9445	0.0195
8	19	0.9604	0.9631	0.9578	0.9604	0.0153
9	88	0.9572	0.9607	0.9528	0.9567	0.0168
10	31	0.9687	0.9709	0.9654	0.9681	0.0134

**Table 18:** Statistical aggregation of results across 10 runs for WUSTL and CICIoMT24 datasets.

Dataset	Statistic	Accuracy	Precision	Recall	F1-score	FPR
WUSTL EHMS 2020	Mean	0.9619	0.9650	0.9579	0.9615	0.0156
	Std. Dev.	0.0151	0.0141	0.0160	0.0151	0.0027
	Aggregate values	0.9619 ± 0.0151	0.9650 ± 0.0141	0.9579 ± 0.0160	0.9615 ± 0.0151	0.0156 ± 0.0027
CICIoMT24	Mean	0.9606	0.9630	0.9579	0.9616	0.0161
	Std. Dev.	0.0151	0.0141	0.0160	0.0151	0.0027
	Aggregate values	0.9619 ± 0.0151	0.9650 ± 0.0141	0.9579 ± 0.0160	0.9615 ± 0.0151	0.0156 ± 0.0027

Mean and standard deviation are reported separately for each metric.

## 6 Conclusion

The need to safeguard healthcare IoT systems using stronger defense mechanisms becomes more significant in the current, technology-driven world, owing to the high sensitivity of the personal healthcare data they contain. Defense mechanisms should address prevention, protection, detection, and effective rollback to a safe state, recovering the system for continued service. This study proposes an effective solution for better privacy and integrity in healthcare IoT systems: a two-tier detection and rollback system. The model enforces deep anomaly detection, analyzing the severity of an attack event and then taking appropriate rollback decisions, choosing the most suitable checkpoints for rollback. Eligible checkpoints undergo post-quantum verification using ML-DSA/CRYSTALS-Dilithium and a combined trust score calculation of the checkpoint's reliability. The post-quantum signature verification also helps to safeguard the checkpoint from future quantum-based adversaries and establish better continuity of system service. This helps return to a recent but trustworthy state so the system can resume operations with minimal downtime, without losing system progress. The model was implemented in the Google Colab environment over two separate datasets widely chosen for security studies on healthcare IoT systems—WUSTL EHMS 2020 and CIC IoMT 2024- and compared with standard baseline models for both detection and recovery. AutoEncoderOnlyDetector, FusionNoRollbackDetector, and MLPOnlyDetector approaches were used as baselines for detection, and LatestCheckpointRollback, NoPolicyRollback, RandomCheckpointRollback, StaticRollback, and StaticThresholdRollback approaches were used as baselines for recovery. The proposed two-tier model was found to outperform these models in all the metrics chosen for evaluation. The model performance was best recorded with 31% attack probability, 97% confidence score, 4.1% anomaly score, 51% severity analysis, 36.23 ns recovery time, 75.3% trust score, 98.19% uptime, 74.33% rollback quality, verification latency of 11.51 ns and 98.19% in F1-score, 98.2% recall, 98.27% precision and false positive rate of 0.012 for WUSTL EHMS 2020 dataset. The model also had 87.45% attack probability, 91% confidence score, 7.11% anomaly score, 88% severity analysis, 16.98 ns recovery time, 76.42% trust score, 98.54% uptime, 71.53% rollback quality, verification latency of 11.05 ns and 98.54% in F1-score, 98.35% recall, 98.51% precision and false positive rate of 0.0146 for CIC IoMT 2024 dataset. The two-tier model thus systematically supports effective and accurate attack detection, severity analysis, and recovery method selection (micro rollback, medium rollback, or complete rollback) based on severity. The post-quantum-based checkpoint verification and selection of checkpoints based on a combined trust score ensures continued service of the system with minimal downtime.

## 7 Future research directions

Future research directions include validation with real-world data, stronger formal security proofs, lightweight edge deployments, compliance and interoperability with healthcare standards, integration of federated learning for checkpoint creation, selection, and validation, and advanced fault-tolerance recovery under diverse attack environments.

## Figures and Tables

**Table 19:** Nomenclature and description.

Symbol	Description
*D*_raw_	Raw input dataset before preprocessing
*v*_*i*_	Feature vector of the *i*-th sample
*l*_*i*_	Label of the *i*-th sample
*N*	Total number of samples in the dataset
*V*_raw_	Raw feature matrix extracted from the dataset
*L*	Original label vector
*E*	Label encoder that maps categorical labels to integers
*l* ^(*m*)^	Multi-class encoded label vector
*l* ^(*b*)^	Binary label vector, where normal traffic is 0 and attack traffic is 1
⊮{·}	Indicator function, equal to 1 if the condition is true and 0 otherwise
normal	Class representing benign or non-attack traffic
*V*_num_	Numeric feature matrix after removing non-numeric columns and imputing missing values
F	Set of retained numeric feature names
*d*	Number of retained numeric features
μ_*j*_	Mean of feature *j*
σ_*j*_	Standard deviation of feature *j*
$v_{ij}^{\text{num}}$	Value of the *j*-th numeric feature for the *i*-th sample before standardization
*v*_*ij*_	Standardized value of the *j*-th feature for the *i*-th sample
*S*	Fitted scaler storing ${\left\{\left(\mu_{j},\sigma_{j}\right)\right\}}_{j=1}^{d}$ for later reuse
*V* ∈ ℝ^*N*×*d*^	Preprocessed feature matrix with *N* samples and *d* features
ρ_min_	Minimum recall floor used during threshold tuning
*f*_θ_	Deep classifier used to estimate attack probability
θ	Trainable parameters of the classifier *f*_θ_
*g*_ϕ_	Autoencoder used to reconstruct normal samples and detect deviations
ϕ	Trainable parameters of the autoencoder *g*_ϕ_
*V*_train_	Training subset of the preprocessed feature matrix
*V*_normal_	Subset of benign samples extracted from the training data
$X_{\text{cls}}$	Binary cross-entropy loss used to train the classifier
$X_{\text{ae}}$	Mean squared reconstruction loss used to train the autoencoder
*P*_atk_(*x*)	Predicted attack probability for input sample *x*
*e*(*v*)	Reconstruction error of sample *v*, measuring deviation from normal behavior
μ_*e*_	Mean reconstruction error computed over normal training samples
σ_*e*_	Standard deviation of reconstruction error computed over normal training samples
ê(*v*)	Normalized reconstruction error mapped to the range [0, 1]
τ_*p*_	Probability threshold used for binary attack prediction
τ_*e*_	Reconstruction-error threshold used for binary attack prediction
α	Fusion weight balancing classifier probability and reconstruction score
*s*(*v*)	Combined anomaly score over attack probability and normalized reconstruction error
$\hat{l}\left(v\right)$	Predicted binary label for sample *v*
*c*(*v*)	Confidence score defined as max{*p*_atk_(*v*), 1−*p*_atk_(*v*)}
Γ(*v*)	Severity score representing the intensity of anomaly or attack risk
ϵ	Small numerical constant added for stability in standard deviation computation
$∥\cdot {∥}_{2}^{2}$	Squared Euclidean norm used to compute reconstruction error

## Data Availability

Publicly available datasets were analyzed in this study. This data can be found here: https://www.cse.wustl.edu/~jain/ehms/index.html, https://www.unb.ca/cic/datasets/iomt-dataset-2024.html.

## Nomenclature

Table 19 summarizes the nomenclature used in various algorithms detailed in this research article.
